# Fut2 Deficiency Promotes Intestinal Stem Cell Aging by Damaging Mitochondrial Functions via Down-Regulating α1,2-Fucosylation of Asah2 and Npc1

**DOI:** 10.34133/research.0343

**Published:** 2024-03-27

**Authors:** Caihan Duan, Zhe Wang, Junhao Wu, Chen Tan, Feifei Fang, Wei Qian, Chaoqun Han, Xiaohua Hou

**Affiliations:** Division of Gastroenterology, Union Hospital, Tongji Medical College, Huazhong University of Science and Technology, Wuhan 430022, China.

## Abstract

Fut2-mediated α1,2-fucosylation is important for gut homeostasis, including the intestinal stem cell (ISC). The stemness of ISC declines with age, and aging-associated ISC dysfunction is closely related to many age-related intestinal diseases. We previously found intestinal epithelial dysfunction in some aged Fut2 knockout mice. However, how Fut2-mediated α1,2-fucosylation affects ISC aging is still unknown. On this basis, the herein study aims to investigate the role of Fut2-mediated α1,2-fucosylation in ISC aging. Aging models in ISC-specific Fut2 knockout mice were established. ISCs were isolated for proteomics and N-glycoproteomics analysis. ISC functions and mitochondrial functions were examined in mice and organoids. Ulex europaeus agglutinin I chromatography and site-directed mutagenesis were used to validate the key target fucosylated proteins of Fut2. As a result, Fut2 knockout impaired ISC stemness and promoted aging marker expression in aged mice. Proteomics analysis indicated mitochondrial dysfunction in Fut2 knockout ISC. More injured mitochondria, elevated levels of reactive oxygen species, and decreased levels of adenosine 5′-triphosphate (ATP) in Fut2 knockout ISC were found. Moreover, respiratory chain complex impairment and mitophagy dysfunction in Fut2 knockout ISC were further noted. Finally, Fut2 was demonstrated to regulate mitochondrial functions mainly by regulating the α1,2-fucosylation of *N*-acyl sphingosine amidohydrolase 2 (Asah2) and Niemann–Pick type C intracellular cholesterol transporter 1 (Npc1). In conclusion, this study demonstrated the substantial role of Fut2 in regulating ISC functions during aging by affecting mitochondrial function. These findings provide novel insights into the molecular mechanisms of ISC aging and therapeutic strategies for age-related intestinal diseases.

## Introduction

The rapid regeneration of intestinal epithelial cells (IECs) that are controlled by intestinal stem cells (ISCs) is critical for maintaining gut homeostasis since the intestinal epithelium is continuously challenged by many damage factors in the lumen [1–3]. However, the functions of IECs decline with age, which leads to damage to the intestinal barrier and loss of intestinal epithelium homeostasis [4]. The key reason is ISC aging, which is characterized by decreased regenerative potential and capacity to generate differentiated cells, also known as “stemness” [5,6]. Impaired ISC function is considered one of the important hallmarks of aging and is closely correlated with many aging-related diseases such as defective absorption of nutrients, chronic inflammation, and carcinoma [7–10]. However, the molecular mechanisms of aging-associated ISC dysfunction remain largely unclear.

Fut2 encodes α1,2-fucosyltransferase, which catalyzes the process of α1,2-fucosylation, a kind of posttranslational modification that covalently links fucose to asparagine of proteins or oligosaccharides in an N-linked manner [11]. α1,2-Fucosylation on IECs is demonstrated to be critical for intestinal epithelial homeostasis [12]. Our previous study revealed that IEC-specific deficiency of Fut2 aggravates colitis and disturbs the gut microbe and metabolism [13]. Furthermore, fucosylation is indispensable for the proper biological function of proteins. For instance, we recently found the fucosylation of the endoplasmic reticulum (ER) protein Hyou1 is important for protecting ISC from excessive ER stress and inflammatory damage in colitis [14]. On this basis, we could reasonably put forward the question of whether Fut2-mediated α1,2-fucosylation affects ISC aging.

Mitochondria are another essential factor in maintaining intestinal epithelium homeostasis [15]. ISCs show high mitochondrial activity, while mitochondrial dysfunction leads to stemness and proliferation damage [16–18]. Importantly, mitochondrial dysfunction is also one of the hallmarks of aging [7,8]. The deficiency of the respiratory chain results in increased electron leakage and reduced adenosine 5′-triphosphate (ATP) generation. Combined with impaired mitochondrial integrity, biogenesis, and mitochondria clearance, these factors have a profound influence on the aging process [8,19]. Interestingly, many mitochondrial proteins are predicted as targets of glycosylation [20]. Although the role of glycosylation in mitochondrial dysfunction is still largely unknown, we could speculate that Fut2-mediated α1,2-fucosylation may participate in regulating mitochondrial functions and therefore affecting ISC aging.

Thus, in this study, we aimed to investigate the role of Fut2-mediated fucosylation in ISC aging. To validate our hypothesis, we examined the expression of Fut2 in aged intestinal crypts first. The effects of Fut2 on ISC aging were explored by generating ISC-specific Fut2 knockout mice and feeding them to 18 months old to establish an aging model. Then, ISCs were isolated for proteomics and N-glycoproteomics analysis to explore the underlying mechanism. The stemness and mitochondrial functions of ISCs were examined, and organoid models were used to verify the supposed mechanisms. Our results indicated the significance of Fut2-mediated α1,2-fucosylation in ISC aging through regulating mitochondrial functions, which provided new insight into stem-cell-based therapeutic strategy for age-associated intestinal diseases.

## Results

### Fut2-mediated fucosylation was reduced in aged human and mouse crypts

**Fig. 1.**
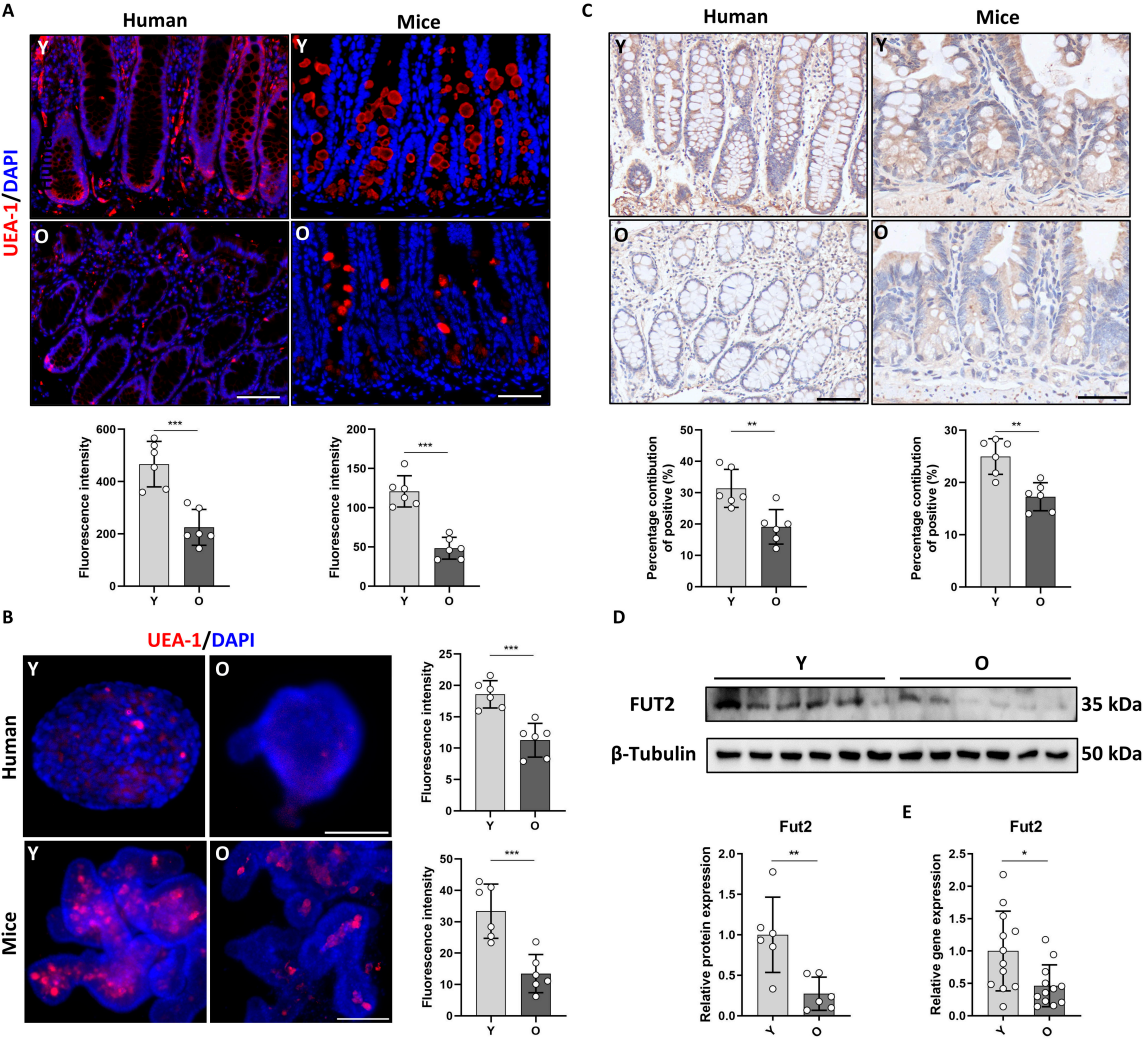
Fut2-mediated fucosylation was reduced in aged crypts. (A) IF analysis of UEA-I staining in young and aged human colon and mouse ileal sections. Scale bars, 100 μm. (B) Representative images of UEA-I staining in organoids derived from young and aged human colon and mouse ileum tissues. Scale bars, 100 μm. (C) IHC analysis of Fut2 expression in young and aged human colon and mouse ileal sections. Scale bars, 100 μm. (D) Western blot analysis of protein expression of Fut2 in young and aged mouse ileal crypts. (E) qPCR analysis of gene expression of Fut2 in young and aged mouse ileal crypts. *P < 0.05, **P < 0.01, and ***P < 0.001.

To investigate the role of Fut2 in ISC aging, we first examined the expression of Fut2 and the level of α1,2-fucosylation in young and aged intestinal specimens from humans and mice. As shown in Fig. 1A, α1,2-fucosylation expression in both human and mouse aged (O) crypts declined compared to young (Y) crypts. In organoids derived from these 2 kinds of crypts, α1,2-fucosylation was also reduced in the aged (Fig. 1B). Immunohistochemistry (IHC) analysis also showed lower expression of Fut2 in aged crypts (Fig. 1C). This result was further supported by Western blot and quantitative polymerase chain reaction (qPCR) analysis that showed lower Fut2 gene and protein expression in aged groups (Fig. 1D and E).

### The stemness of ISCs was decreased more in aged Fut2-deficient mice

**Fig. 2.**
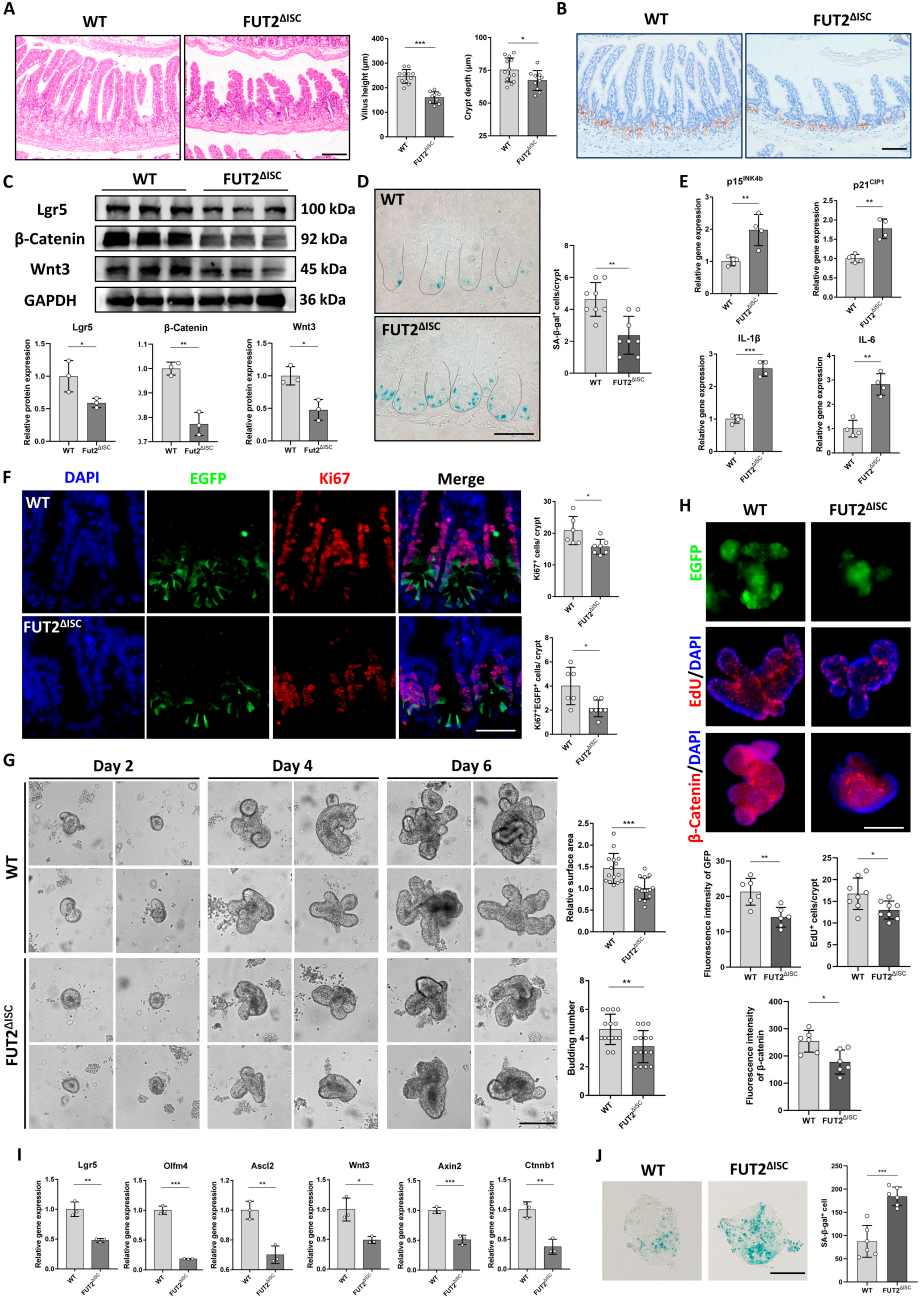
Fut2 deficiency suppresses the stemness of ISCs in aging mice. (A) Left: H&E staining of ileal sections from aged WT and Fut2^ΔISC^ mice. Scale bar, 100 μm. Right: Statistical analysis of villus height and crypt depth of ileum. (B) IHC results of Olfm4 in ileal sections from aged WT and Fut2^ΔISC^ mice. Scale bar, 100 μm. (C) Protein expression of Lgr5, Wnt3, and β-catenin in crypts of aged WT and Fut2^ΔISC^ mice. (D) SA-β-gal staining in ileal sections from aged WT and Fut2^ΔISC^ mice. Blue staining indicates senescence cells, and the dotted line indicates crypt. Scale bar, 100 μm. (E) Gene expression of senescence markers and senescence-associated secretory phenotypes in crypts of aged WT and Fut2^ΔISC^ mice. (F) EGFP and Ki67 costaining in ileal sections from aged WT and Fut2^ΔISC^ mice. Scale bar, 100 μm. (G) Development of organoids derived from WT and Fut2^ΔISC^ mice and statistical analysis of surface area and budding number. Scale bar, 100 μm. (H) IF analysis of EGFP, EdU, and β-catenin in organoids derived from aged WT and Fut2^ΔISC^ mice. Scale bar, 100 μm. (I) Gene expression of stemness markers in WT and Fut2^ΔISC^ organoids derived from aged WT and Fut2^ΔISC^ mice. (J) SA-β-gal staining in WT and Fut2^ΔISC^ organoids derived from aged WT and Fut2^ΔISC^ mice. Scale bar, 100 μm. *P < 0.05, **P < 0.01, and ***P < 0.001.

The role of Fut2 in ISC aging was further investigated by establishing an aging mouse model in ISC-specific Fut2-deficient mice. We fed wild-type (WT) and Fut2^ΔISC^ mice to 18 months old and examined the histological alteration in these 2 kinds of mice. Hematoxylin and eosin (H&E) staining showed that the villus heights and crypt depth in Fut2^ΔISC^ mice declined more than those in WT mice, which indicates the atrophy of the intestine and decreased functions of ISCs (Fig. 2A). Since the number and proliferation potential of ISCs were decreased while aging and one of the causes is the reduced canonical Wnt signaling, we examined these phenotypes in mice [21]. As a result, there were fewer olfactomedin 4 (Olfm4)-indicated ISCs in the ileal crypt of Fut2^ΔISC^ mice, and the protein expression of ISC markers leucine rich repeat containing G protein-coupled receptor 5 (Lgr5) in the ileal crypts of Fut2^ΔISC^ mice was also decreased compared to WT (Fig. 2B and C). A reduced Wnt signaling was also found in crypts of Fut2^ΔISC^ mice, indicated by the reduced protein expression of β-catenin and Wnt3 (Fig. 2C and Fig. S2A). We further examined the senescence marker senescence β-galactosidase (SA-β-gal) and found more SA-β-gal staining in the crypts of Fut2^ΔISC^ mice (Fig. 2D). The expression of senescence marker genes p15^INK4b^, p21^CIP1^, interleukin-1β (IL-1β), and IL-6, which are senescence-associated secretory phenotypes, was increased in Fut2^ΔISC^ mice compared to WT mice (Fig. 2E). Moreover, the proliferation was indicated by Ki67 and enhanced green fluorescent protein (EGFP) staining. The counts of Ki67^+^ cells and Ki67^+^EGFP^+^ cells were both fewer in Fut2^ΔISC^ mice (Fig. 2F), indicating less proliferation activity of Fut2-deficient ISCs.

To further study the influence of Fut2 deficiency on the functions of ISCs, we derived organoids from the ileum of 18-month-old WT and Fut2^ΔISC^ mice since the 3-dimensional organoid culture can mimic the in vivo physiology of the intestinal epithelium and it can therefore be used as a tool for intestinal developmental and regenerative investigation [22]. As shown in Fig. 2G, the organoid formation and development in Fut2-deficient organoids were restrained compared to the WT group, indicated by the decreased surface area and number of buds. The counts of EGFP^+^ ISCs and 5-ethynyl-2′-deoxyuridine (EdU^+^) proliferation cells, as well as the β-catenin expression in organoids, were also reduced in Fut2^ΔISC^ organoids (Fig. 2H). The results of real-time PCR also showed a decreased expression of ISC markers and Wnt signaling genes Lgr5, Olfm4, Ascl2, Wnt3, Axin2, and Ctnnb1 (Fig. 2I). In addition, more SA-β-gal staining was found in Fut2^ΔISC^ organoids (Fig. 2J). Collectively, these results indicated that Fut2 deficiency in ISC promoted its senescence and suppressed its stemness.

### Fut2 deficiency of ISCs promoted the d-galactose-induced senescence in murine gut

We next established a d-galactose (d-gal)-induced accelerative senescence model in adult WT and Fut2^ΔISC^ mice to further confirm the effects of Fut2 in ISC aging. There were some similar alterations with the results in natural aging mice, such as decreased villus heights and crypt depth, more SA-β-gal staining in crypts, and increased levels of p15^INK4b^, p21^CIP1^, IL-1β, and IL-6 expression in Fut2^ΔISC^ + d-gal mice compared with WT + d-gal group (Fig. S3A to C). Since the d-gal induces oxidative stress to promote senescence, we examined levels of oxidative-stress-related enzymes catalase (CAT), glutathione peroxidase (GSH-PX), malondialdehyde (MDA), and superoxide dismutase (SOD) in crypts. The levels of CAT, GSH-PX, and SOD decreased more in crypts of Fut2^ΔISC^ mice after d-gal treatment compared to WT mice, while MDA increased more, which indicated more serious oxidative-stress-induced damage in crypts of Fut2^ΔISC^ + d-gal group (Fig. S3D). Olfm4 staining showed that the ISC number of the Fut2^ΔISC^ + d-gal mice was less than the WT + d-gal mice (Fig. S3E).

Then, we derived organoids from adult WT and Fut2^ΔISC^ mice and treated them with H_2_O_2_ to induce oxidative stress damage, which is a widely used method to induce cell aging [23]. EGFP staining showed less Lgr5 expression in Fut2^ΔISC^ organoids after H_2_O_2_ treatment, and the proliferation was decreased more compared to WT (Fig. S3F and G). Meanwhile, SA-β-gal staining showed more senescent cells in the Fut2^ΔISC^ + H_2_O_2_ group, and the expression of p15^INK4b^, p21^CIP1^, IL-1β, and IL-6 was raised (Fig. S3H and I).

Moreover, we found that the tight junction of intestinal epithelium in Fut2^ΔISC^ mice and organoids received more serious injury after oxidative stress damage, as shown by ZO-1 and Claudin-1 staining (Fig. S4A and B), and more apoptosis was found in Fut2^ΔISC^ + H_2_O_2_ organoids that were indicated by cleaved caspase 3 staining (Fig. S4C). Furthermore, the counts of proliferation cells in crypts of Fut2^ΔISC^ + d-gal mice were also decreased (Fig. S4D). These results showed that Fut2 deficiency makes ISCs more vulnerable to oxidative-stress-induced aging.

### Mitochondrial functions were injured in aged Fut2-deficient ISCs

**Fig. 3.**
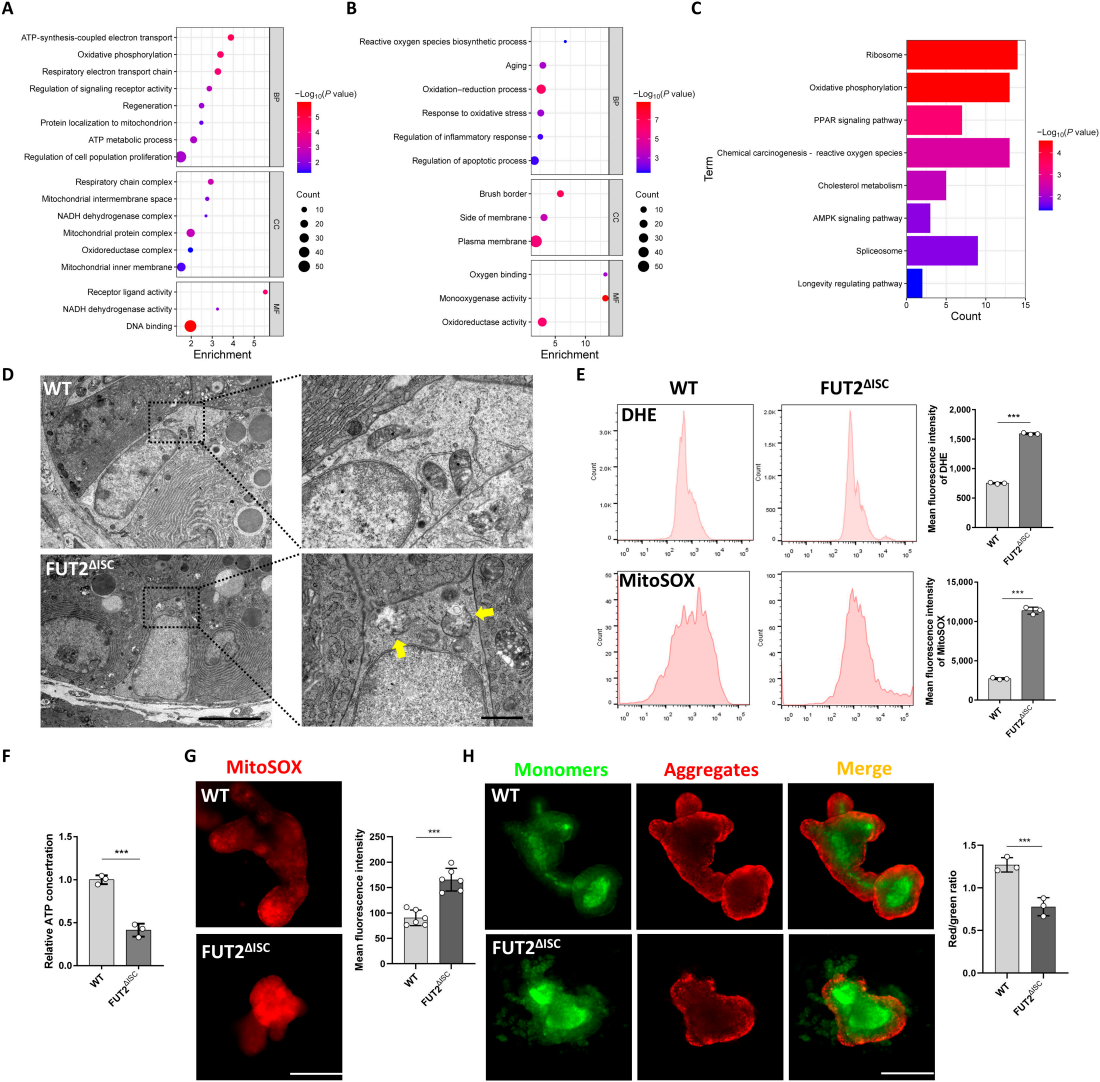
Fut2 deficiency injured mitochondria in ISCs. (A) GO enrichment analysis of down-regulated expressed proteins in ISCs of WT and Fut2^ΔISC^ mice. NADH, reduced form of nicotinamide adenine dinucleotide (oxidized form). BP, biological process; CC, cellular component; MF, molecular function. (B) GO enrichment analysis of up-regulated expressed proteins in ISCs of WT and Fut2^ΔISC^ mice. (C) KEGG enrichment analysis of differently expressed proteins in ISCs of WT and FUT2^ΔISC^ mice. PPAR, peroxisome-proliferator-activated receptor; AMPK, adenosine 5′-monophosphate-activated protein kinase. (D) TEM analysis of mitochondria in ISCs of aged WT and Fut2^ΔISC^ mice. Yellow arrows indicate injured mitochondria. Scale bars, 5 and 1 μm. (E) Flow cytometry analysis of ROS and mtROS level in ISCs of aged WT and Fut2^ΔISC^ mice with DHE and MitoSOX probes, respectively. (F) Detection of ATP level in crypts of aged WT and Fut2^ΔISC^ mice. (G) MitoSOX-indicated mtROS detected by fluorescence microscope in organoids derived from aged WT and Fut2^ΔISC^ organoids. Scale bar, 100 μm. (H) MMP in WT and Fut2^ΔISC^ organoids derived from aged mice. Scale bar, 100 μm. ***P < 0.001.

We next sorted WT and Fut2 knockout ISCs by flow sorter and performed proteomics analysis to investigate the probable ways through which Fut2 deficiency promoted ISC aging. Analysis in differential expression proteins showed that Gene Ontology (GO) annotation terms that related to mitochondrial functions such as ATP synthesis, protein localization to the mitochondrion and respiratory chain complex, etc. were down-regulated, while reactive oxygen species (ROS) biosynthesis, aging, and oxidative stress were up-regulated in Fut2-deficient ISCs compared to WT group (Fig. 3A and B). Kyoto Encyclopedia of Genes and Genomes (KEGG) pathway analysis also showed alteration of oxidative phosphorylation, ROS, and longevity regulating pathway in Fut2 knockout ISCs (Fig. 3C). On the basis of the results described above, we hypothesized that Fut2 may affect ISC aging by regulating mitochondrial functions. Therefore, we next intended to detect mitochondrial functions in WT and Fut2 knockout ISCs. Transmission electron microscope (TEM) analysis showed more severe damage of mitochondria in ISCs of senescence Fut2^ΔISC^ mice such as mitochondrial swelling and cristae fracture (Fig. 3D and Fig. S5A), which was accompanied by increased ROS/mitochondrial ROS (mtROS) levels that were respectively indicated by dihydroethidium (DHE) and MitoSOX probes and decreased ATP level (Fig. 3E and F and Fig. S5B to E). In organoids derived from aging Fut2^ΔISC^ mice, the MitoSOX-indicated mtROS level in Fut2^ΔISC^ organoids was higher than that in WT (Fig. 3G). We further detected the mitochondrial membrane potential (MMP) with JC-1 fluorescent probe. We found that the MMP was decreased in Fut2^ΔISC^ organoids compared to WT, which was indicated by decreased JC-1 aggregates/monomers (Fig. 3H and Fig. S5F). Furthermore, we also found more fragmented mitochondria in Fut2 knockout ISCs compared to the WT, as shown by Tomm20 staining (Fig. S3G).

### Dysfunctional respiratory chain complexes and mitophagy were found in Fut2-deficient ISCs

To further explore the reasons for mitochondria damage caused by Fut2 deficiency in ISCs, we first detected mitochondrial DNA and mitochondrial marker proteins Tomm20, Timm23, and Vdac1 and noted no significant differences between these 2 groups. Moreover, expressions of mitochondrial dynamics proteins PGC1α, Mfn1, Mfn2, Drp1, and Tfam remained unaltered (Fig. S6A to C). These results indicated that the mitochondria damage may not result from alterations in mitochondrial content and dynamics.

**Fig. 4.**
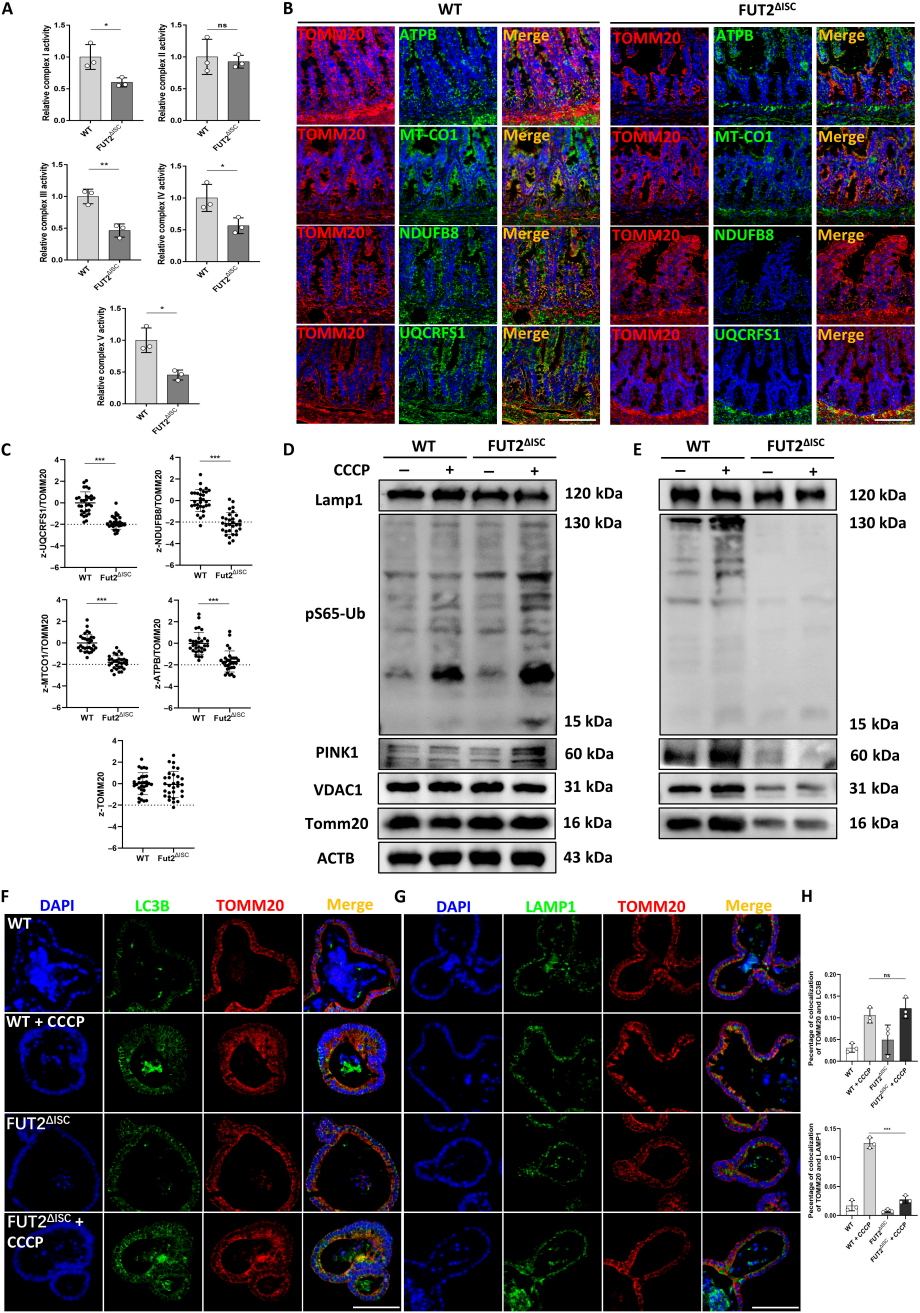
Fut2 deficiency in ISCs induced respiratory chain complex damage and mitophagy dysfunction. (A) Analysis of the activity of complexes I to V in ISCs of WT and Fut2^ΔISC^ mice. (B) IF analysis of costaining of complexes I (Uqcrfs1), III (Ndufb8), IV (Mt-co1), and V (Atpb) marker proteins and mitochondrial marker protein Tomm20. Scale bars, 100 μm. (C) *z* score of the relative activity of complexes I, III, IV, and V. (D) Representative images of mitochondrial and mitophagy protein expression in whole-cell lysate. CCCP was used to activate mitophagy. (E) Mitochondrial and mitophagy protein expression in lysosome fraction. (F to H) IF costaining of LC3B, Tomm20, and Lamp1 in WT and Fut2^ΔISC^ organoids derived from aged mice and statistical analysis of colocalization. Scale bars, 100 μm. *P < 0.05, **P < 0.01, and ***P < 0.001. ns, not significant.

We then detected the conditions of the respiratory chain complexes, which were significantly enriched in proteomics analysis. Results showed the activity of complexes I, III, IV, and V declined in ISCs of Fut2^ΔISC^ mice compared to the WT group (Fig. 4A). We used immunofluorescence (IF) analysis of respiratory chain complexes marker proteins and *z* scores to further verify these results referenced to previous studies [24,25]. As shown in Fig. 4B and C, although there was no difference in the *z* score of Tomm20 between crypts of aged WT and Fut2^ΔISC^ mice, *z* scores of complex proteins Ndufb8 (complex I), Uqcrfs1 (complex III), MT-CO1 (complex IV), and Atpb (complex V) relative to Tomm20 were significantly decreased, indicating deficiencies in respiratory complexes but not mitochondrial mass in Fut2^ΔISC^ mice.

Mitophagy eliminates damaged mitochondria and controls the quality of mitochondria by transporting dysfunctional mitochondria to lysosomes for degradation in vesicles and impaired mitophagy results in the accumulation of dysfunctional mitochondria [26]. We speculated whether Fut2 deficiency may also induce mitophagy impairment. Therefore, we next investigated the mitophagy in organoids derived from aging WT and Fut2^ΔISC^ mice. Mitophagy activator carbonyl cyanide 3-chlorophenylhydrazone (CCCP) treatment activated the mitophagy process in both WT and Fut2^ΔISC^ organoids, as indicated by the increased expression of phosphatase and tensin homolog induced kinase 1 (PINK1) and phospho-ubiquitin-S65 (pS65-Ub) (Fig. 4D). However, we further examined these mitophagy-related proteins, as well as mitochondrial proteins Tomm20 and Vdac1, in lysosome fraction proteins and found that there was no correspondingly increased expression of these proteins (Fig. 4E) in Fut2^ΔISC^ organoids that treated with CCCP, which may indicate a lysosome-deficiency-induced mitophagy impairment resulted in Fut2 knockout. Accordant with these, immunostaining showed increased colocalization of autophagy marker LC3B and mitochondrial marker Tomm20 but not correspondingly increased colocalization of lysosome marker Lamp1 and Tomm20 after CCCP treatment in Fut2^ΔISC^ organoids (Fig. 4F to H).

In summary, these results indicated that Fut2 deficiency in ISC impaired mitochondrial functions mainly by injuring mitochondrial respiratory complexes and mitophagy.

### α1,2-Fucosylation of mitochondrial-function-related proteins was impaired in Fut2-deficient ISCs

**Fig. 5.**
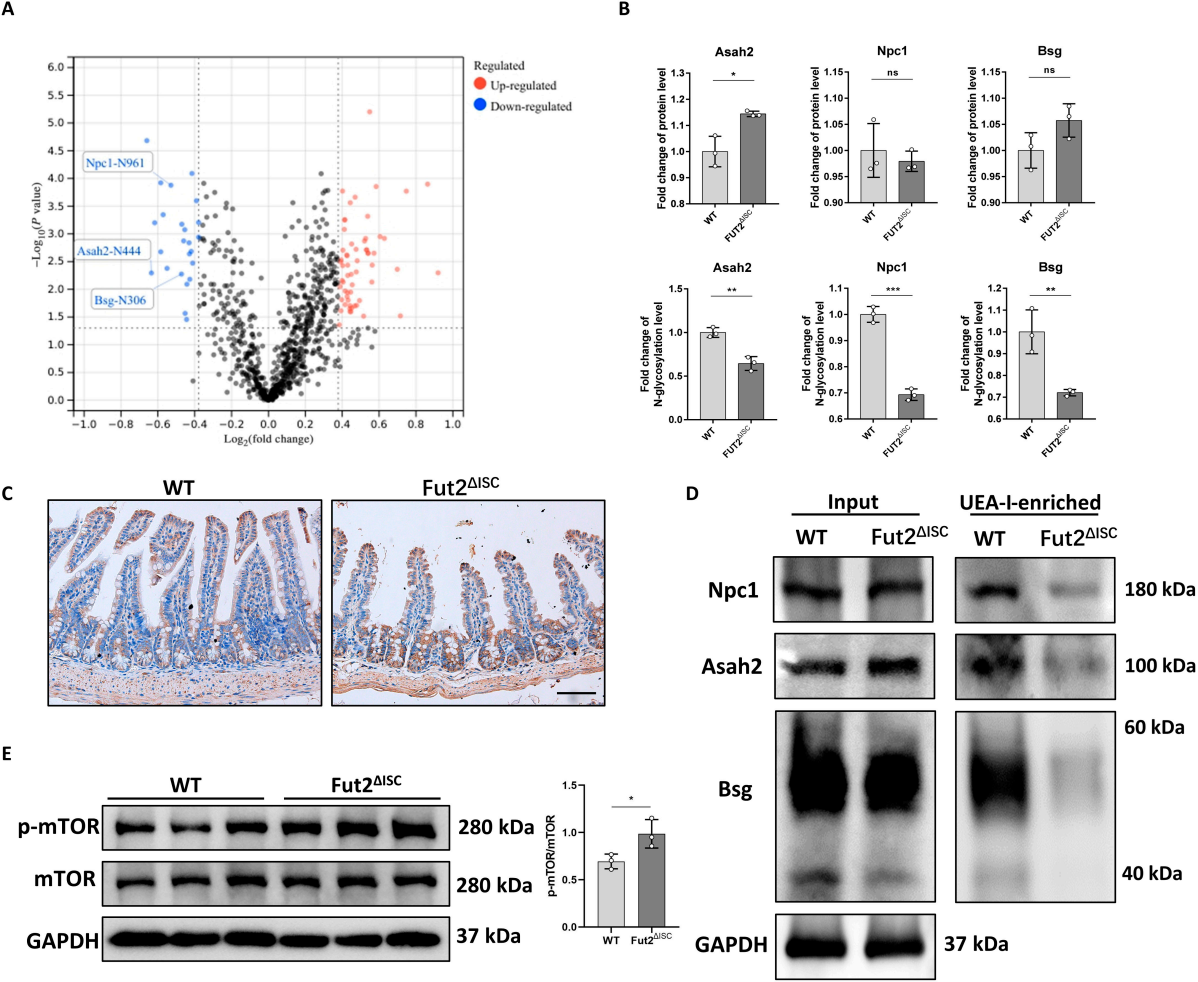
Fut2 deficiency in ISCs resulted in impairment of α1,2-fucosylation of mitochondrial-function-related proteins. (A) Volcano plot of differently expressed N-glycosylated proteins and sites in Fut2 knockout ISCs compared to WT. (B) Fold change of protein level and N-glycosylation level of mitochondrial-function-related proteins Asah2, Npc1, and Bsg. (C) IHC analysis of Asah2 in ileal sections of WT and Fut2^ΔISC^ mice. Scale bar, 100 μm. (D) The protein level of Npc1, Asah2, and Bsg in whole-cell lysate and UEA-I-enriched proteins of WT and Fut2^ΔISC^ ISCs. (E) Protein expression of mTOR and p-mTOR in WT and Fut2^ΔISC^ crypts. *P < 0.05, **P < 0.01, and ***P < 0.001. ns, no significant.

The direct effect of Fut2 is to mediate the α1,2-fucosylation modification of glycoproteins, so we next investigate whether Fut2 deficiency affected the mitochondrial function through regulating the α1,2-fucosylation modification of specific mitochondrial-function-related proteins. We performed N-linked glycosylation modification proteomics to detect the alteration of N-glycosylation modification in Fut2 knockout ISC, and the results showed that there were 23 down-regulated proteins with 24 N-glycosylation sites and 47 up-regulated proteins with 60 sites in Fut2 knockout ISC compared to WT. Among the down-regulated proteins, we found 3 proteins that were related to mitochondrial functions: *N*-acyl sphingosine amidohydrolase 2 (Asah2), Niemann–Pick type C intracellular cholesterol transporter 1 (Npc1), and basigin (Bsg) (Fig. 5A). The fold change of N-glycosylation level of Asah2, Npc1, and Bsg is 0.644, 0.693, and 0.721, respectively (Fig. 5B). Asah2 (encodes neutral ceramidase) functioned to hydrolyze sphingolipid ceramides, and abnormality of Asah2 would induce accumulation of ceramides, which has been demonstrated to injure the respiratory chain complexes and induce mitochondrial dysfunction [27,28]. However, the role of Asah2 in ISC has not been reported before. We examined Asah2 expression by IHC staining and found that there was abundant expression of it in crypts (Fig. 5C). Npc1 is a lysosome membrane protein, and loss of Npc1 causes mitophagy dysfunction [29]. Bsg was also reported to relate to mitochondrial energy metabolism [30]. We then verified whether the decreased N-glycosylation on these proteins is a consequence of α1,2-fucosylation deficiency. Since ulex europaeus agglutinin I (UEA-I) could combined with α1,2-fucosylated proteins, we could enrich α1,2-fucosylated proteins from cell lysate using an agarose-bound UEA-I. As shown in Fig. 5D, although the global expression of Asah2, Npc1, and Bsg did not significantly alter between WT and Fut2^ΔISC^ crypts, the expression of these 3 proteins in UEA-I-enriched protein was significantly decreased in Fut2^ΔISC^ group, which indicated that the α1,2-fucosylation modification on Asah2, Npc1, and Bsg was reduced because of Fut2 deficiency. In addition, mammalian target of rapamycin (mTOR), the downstream target of Npc1, was activated in Fut2^ΔISC^ crypts (Fig. 5E).

### Loss of α1,2-fucosylation of Asah2 and Npc1 induced stemness impairment and mitochondrial dysfunction in ISCs

**Fig. 6.**
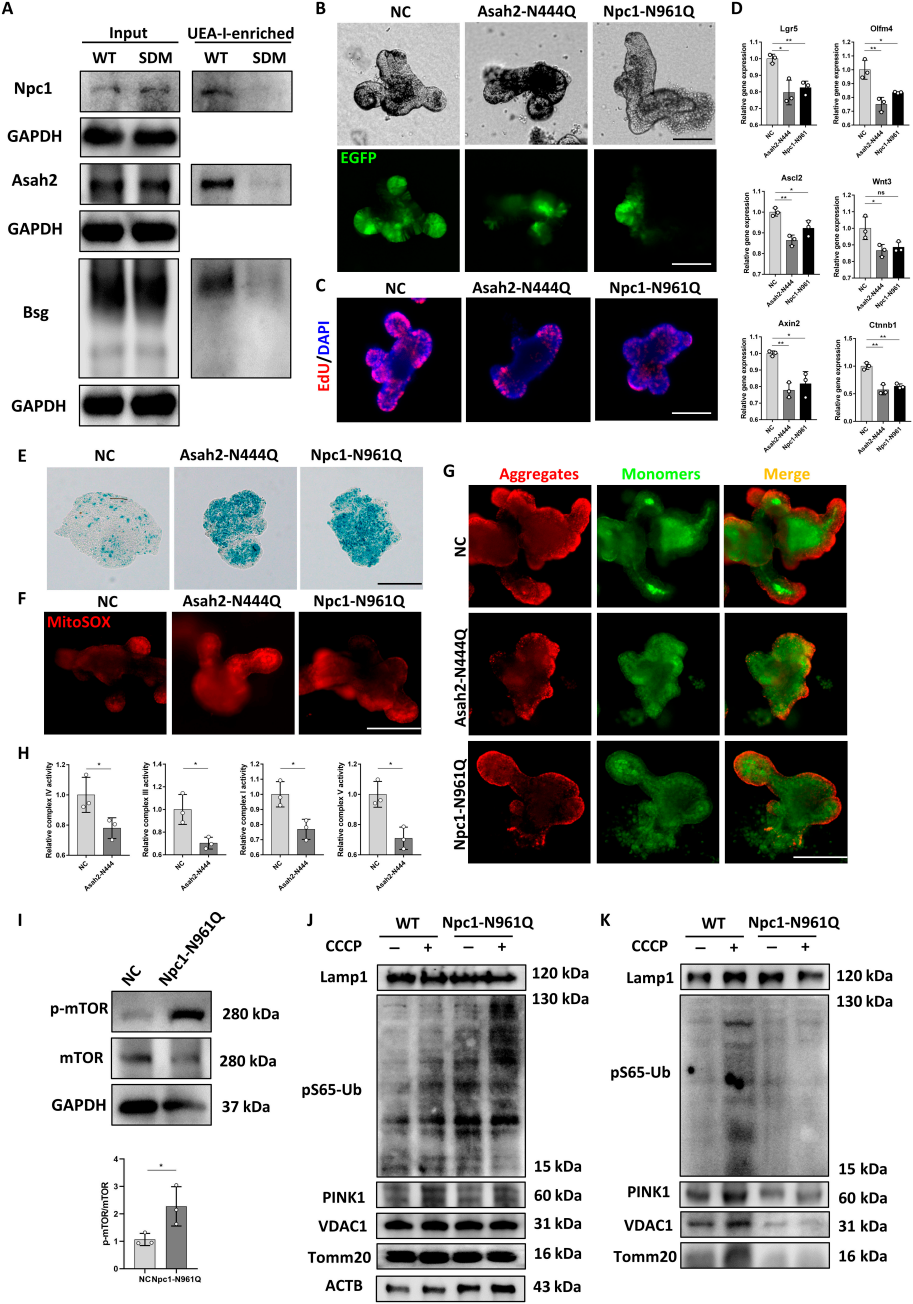
Loss of α1,2-fucosylation of Asah2 and Npc1 induced stemness impairment and mitochondrial dysfunction in ISCs. (A) The protein level of Npc1, Asah2, and Bsg in whole-cell lysate and UEA-I-enriched proteins of WT and corresponding SDM organoids. (B) Images of WT, Asah2-N444Q, and Npc1-N961Q organoids and IF analysis of EGFP-labeled ISCs. Scale bars, 100 μm. (C) EdU assays in WT, Asah2-N444Q, and Npc1-N961Q organoids. Scale bar, 100 μm. (D) qPCR results of stemness markers in WT, Asah2-N444Q, and Npc1-N961Q organoids. (E to G) SA-β-gal staining of senescent cells, MitoSOX-indicated mtROS, and JC-1-indicated MMP analysis in WT, Asah2-N444Q, and Npc1-N961Q organoids. Scale bars, 100 μm. (H) Activity assays of respiratory complexes I, III, IV, and V in WT and Asah2-N444Q organoids. (I) Protein expression of mTOR and p-mTOR in WT and Npc1-N961Q organoids. (J) Expression of mitochondrial and mitophagy proteins in the whole-cell lysate of WT and Npc1-N961Q organoids. CCCP was used to activate mitophagy. (K) Expression of mitochondrial and mitophagy proteins in lysosome fractions of WT and Npc1-N961Q organoids. *P < 0.05 and **P < 0.01.

As Fut2 deficiency was demonstrated to induce loss of α1,2-fucosylation modification on Asah2, Npc1, and Bsg, we would like to validate further whether the decreased fucosylation of Asah2, Npc1, and Bsg could impair the stemness and mitochondrial functions in ISCs. As mentioned above, α1,2-fucosylation occurs at the asparagine of proteins. When the asparagine site is mutant, the fucosylation will be inhibited. Therefore, we used the site-directed mutagenesis (SDM) method to generate Asah2, Npc1, and Bsg mutants to mutate the selective asparagine site to glutamine. According to the results of N-glycoproteomics, we mutate asparagine-444 (N444) of Asah2, N961 of Npc1, and N306 of Bsg, the fucosylation site of these 3 proteins, to glutamine (Q), respectively, which prevented the attachment of fucose to the amino acids (abbreviated to Asah2-N444Q, Npc1-N961Q, and Bsg-N306Q). To validate the efficiency of mutation, we examined α1,2-fucosylation on Asah2, Npc1, and Bsg in WT and SDM groups by UEA-I chromatography first. As a result, the α1,2-fucosylation of Asah2, Npc1, and Bsg was respectively decreased in these SDM organoids (Fig. 6A). The EGFP-Lgr5 expression and EdU-labeled proliferation cells in Asah2-N444Q and Npc1-N961Q organoids were reduced compared to the negative control (NC) group (Fig. 6B and C), along with reduced expression of ISC marker genes and Wnt signaling pathway genes (Fig. 6D). However, the mutant of Bsg-N306Q seemed to show no significant effects on the stemness of ISCs (Fig. S7A to C). We speculate that only the decrease in Fut2-mediated α1,2-fucosylation of Bsg may not be sufficient enough to induce the impairment of mitochondrial function and stemness in ISC. Moreover, increased SA-β-gal staining was also detected in Asah2-N444Q and Npc1-N961Q organoids compared to the NC group (Fig. 6E). We further observed increased MitoSOX-staining-indicated mtROS levels and decreased MMP indicated by decreased JC-1 aggregates in Asah2-N444Q and Npc1-N961Q but not in Bsg-N306Q (Fig. 6F and G and Fig. S7D and E). Therefore, we considered Asah2 and Npc1 as the key fucosylated proteins that were regulated by Fut2 in the current study. Since Asah2 was reported to affect the respiratory chain and Npc1 could influence mitophagy, we examined the respiratory chain complex activity in Asah2-N444Q organoids and the state of mitophagy in Npc1-N961Q organoids. As shown in Fig. 6H, the activity of complexes I, III, IV, and V was reduced in Asah2-N444Q organoids, and the mTOR was activated in Npc1-N961Q organoids (Fig. 6I). In addition, similar to that in Fut2^ΔISC^ organoids, the mitophagy could be activated by CCCP in Npc1-N961Q organoids (Fig. 6J), which was indicated by increased expression of Pink1 and pS65-Ub. However, the mitophagy and mitochondrial proteins were not increased correspondingly in the lysosome fractions of Npc1-N961Q organoids treated by CCCP (Fig. 6K), which was also similar to the results of Fut2 deficiency. These results indicated the defective mitophagy in Npc1-N961Q organoids.

Put it all together, we considered that loss of α1,2-fucosylation in Asah2 would induce respiratory chain complex damage and loss of α1,2-fucosylation in Npc1 would induce mitophagy dysfunction in ISCs and therefore lead to impairment of stemness and promote aging.

### Asah2 supplement and mTOR inhibition ameliorated stemness impairment and mitochondrial dysfunction in Fut2^ΔISC^ organoid

**Fig. 7.**
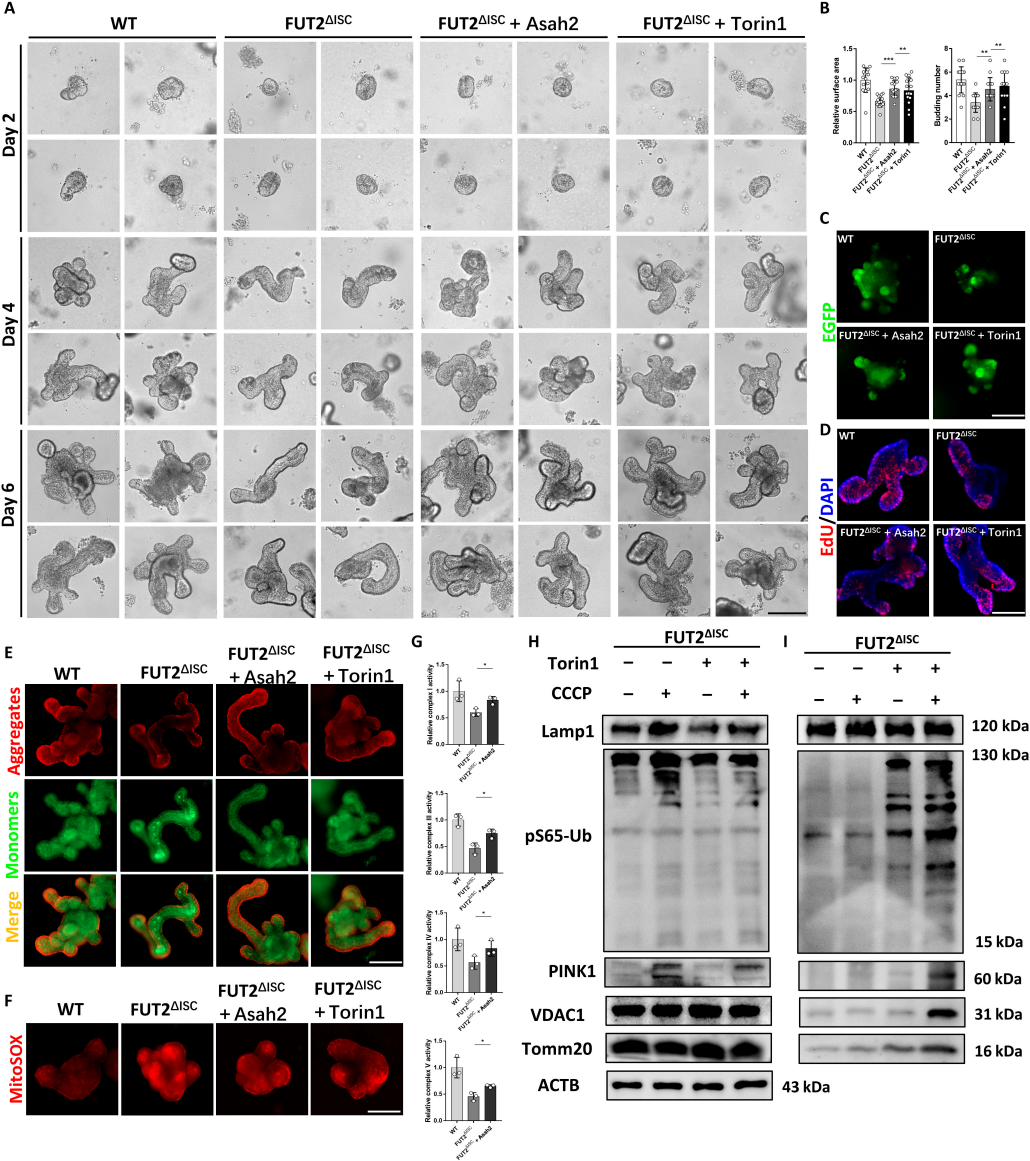
Asah2 supplement and mTOR inhibition ameliorated stemness impairment and mitochondrial dysfunction in Fut2^ΔISC^ organoids. (A) Development of organoids derived from WT and Fut2^ΔISC^ mice, with or without Asah2 and Torin1 treatment. Scale bar, 100 μm. (B) Statistical analysis of surface areas and budding number of organoids. (C and D) IF analysis of EGFP and EdU in WT and Fut2^ΔISC^ organoids, with or without Asah2 and Torin1 treatment. Scale bars, 100 μm. (E and F) JC-1-indicated MMP and MitoSOX-indicated mtROS analysis in WT and Fut2^ΔISC^ organoids, with or without Asah2 and Torin1 treatment. Scale bars, 100 μm. (G) Activity of complexes I, III. IV, and V in WT, Fut2^ΔISC,^ and Fut2^ΔISC^ + Asah2 organoids. (H) Expression of mitochondrial and mitophagy proteins in whole-cell lysate of Fut2^ΔISC^ and Fut2^ΔISC^ + Torin1 organoids. CCCP was used to activate mitophagy. (I) Expression of mitochondrial and mitophagy proteins in lysosome fractions of Fut2^ΔISC^ and Fut2^ΔISC^ + Torin1 organoids. *P < 0.05, **P < 0.01, and ***P < 0.001.

Finally, we performed rescue experiments to further validate the role of Asah2 and Npc1 in Fut2-deficiency-induced mitochondrial dysfunction and stemness impairment. Recombination Asah2 protein and mTOR inhibitor Torin1 were used to treat the Fut2^ΔISC^ organoids to respectively rescue the functions of Asah2 and Npc1 as previously reported [29,31]. The results suggested that the development of organoids in the Fut2^ΔISC^ + Asah2 and FUT2^ΔISC^ + Torin1 organoids was improved compared to the Fut2^ΔISC^ group (Fig. 7A and B). The number of EGFP-labeled Lgr5^+^ ISCs and EdU-labeled proliferation cells was also increased in these 2 groups (Fig. 7C and D), and the MMP was elevated, suggested by increased JC-1 aggregates, while the mtROS level was decreased, suggested by decreased MitoSOX staining, after Asah2 and Torin1 treatment (Fig. 7E and F). Furthermore, the activity of mitochondrial respiratory chain complexes was higher in Fut2^ΔISC^ + Asah2 organoids than Fut2^ΔISC^ organoids (Fig. 7G), and Torin1 treatment restored the process of mitophagy in Fut2-deficient organoids, as indicated by the increased expression of mitophagy and mitochondrial proteins both in whole-cell proteins and in lysosome fractions of Fut2^ΔISC^ + Torin1 organoids under CCCP treatment (Fig. 7H and I).

Collectively, these results demonstrated that Fut2-deficiency-induced loss of α1,2-fucosylation of Asah2 and Npc1 in ISCs accelerated the mitochondrial-dysfunction-mediated aging.

## Discussion

Aging has been involved in many chronic intestinal disorders, such as malabsorption and inflammation. However, our understanding of the mechanism of ISC aging and the intrinsic alteration of ISC during aging remains limited. In the current study, the critical role of Fut2-mediated α1,2-fucosylation in ISC aging was noted. The expression of Fut2 was decreased in senescence ISCs, and Fut2 deficiency impaired the stemness of ISC in aging mice through inducing mitochondrial dysfunction. Notably, through N-glycosylation modification proteomics analysis and organoid model, we demonstrated Asah2 and Npc1 as the key targets of Fut2 in affecting the mitochondrial function of senescence ISC.

Although accumulating research aimed to elucidate the composition and function alteration in the aging gut, our knowledge concerning intrinsic alteration that drives aging-related ISC dysfunction is still incomplete, especially in mammals [6]. Here, we reported that Fut2 expression was reduced in senescence ISCs and indicated its facilitation effects on ISC aging. Although the definite reasons are not so clear yet, possible assumptions could be put forward. Aging-related molecular alteration in ISC is induced by extrinsic and intrinsic factors. In the ISC niche, the microbiome and immune cells are important components [32,33]. Interestingly, intestinal epithelial Fut2 can be regulated by microbes and mucosal immune cells. For instance, the adult microbiota was demonstrated to be necessary for Fut2 induction, and *Bacteroides thetaiotaomicron*, *Bacteroides fragilis*, and segmented filamentous bacteria (SFB) could induce the α1,2-fucosylation on IEC. Group 3 innate lymphoid cells produce IL-22 to activate the IL-22R-signal transducers and activators of transcription 3 signaling to induce Fut2 expression in IECs. The interaction between microbes, fucosylation, and immune cells maintains the gut homeostasis [12,34,35]. As we know, microbial dysbiosis and decline in immune responses occur with age, and dysbiosis may also be involved in reshaping immune responses during aging [36,37]. These aging-related dysfunctions of microbiome and immune cells may contribute to the down-regulation of Fut2 in aging ISC. Moreover, there are also associations among the intestinal-crypt-resided biofilms and epithelial-Fut2-mediated fucosylation [38], whose disorder with age may be another important reason for Fut2 deficiency in senescence ISC.

The role of fucosylation in intestinal senescence has been little explored until now. Defective Fut2 was demonstrated to be associated with several kinds of intestinal diseases, especially colitis. This may be attributed to dysbiosis and dysfunction of metabolism induced by Fut2 deficiency [39]. We previously reported that loss of Fut2-mediated fucosylation in IECs makes them susceptible to inflammatory injury [40]. In addition, epithelial Fut2 deficiency induces dysbiosis of gut microorganisms and dysfunction of the intestinal epithelial barrier [13,41]. Importantly, loss of intestinal barrier function is associated with ISC dysfunction in the aging gut. The epithelial barrier dysfunction is correlated to aging-related inflammatory markers and could be the perspective of death of *Drosophila* [6,42]. In this study, we also found aggravated epithelial barrier disruption in the oxidative-stress-induced aging model in Fut2^ΔISC^ mice and organoids compared to the WT group, which may be an important contributor to Fut2-deficiency-induced stemness impairment and aging of ISC. As we know, the barrier dysfunction could lead to dysbiosis, which may exacerbate the deficiency of Fut2-mediated fucosylation and set up a vicious circle [43]. Furthermore, our previously study found that Fut2 deficiency in IECs promote colorectal cancer (CRC) progression in a dextran sulfate sodium/azoxymethane (DSS/AOM) mouse model [44]. Given the strong connection between dysfunctional ISCs and cancer development [45], whether Fut2 deficiency in ISCs contributes to the pathogenesis of CRC during aging may be a meaningful question in future studies.

We further explored the underlying mechanism by which Fut2 deficiency promotes ISC aging. Through proteomics analysis, we found that Fut2 deficiency mainly resulted in mitochondrial dysfunction in aging ISC. As pivotal regulators of intestinal epithelial homeostasis, multiple aspects of mitochondrial biological processes are critical for maintaining ISC function, and mitochondrial dysfunction is also one of the markers of stem cell aging [8,15,46]. On the one hand, mitochondrial energy metabolisms such as pyruvate metabolism restrict the proliferation of ISCs [47]. On the other hand, mitochondrial quality control systems such as biogenesis, fusion, fission, and mitophagy control the fate of ISC [17]. In addition, there is a bidirectional cross-talk between mitochondria and Wnt signaling, which is the most important signaling pathway in ISC function regulation [48]. Although the crucial role of posttranslational modifications in regulating mitochondrial functions was gradually demonstrated, few studies focused on glycosylation, especially fucosylation [20,49]. Fut2 seemed to mainly affect respiratory chain complexes and mitophagy as described in our study, which are closely associated with aging-related diseases as previously reported [50]. Highly active mitochondrial metabolism and a sufficient number of healthy mitochondria support the stemness of ISCs, while their disorders impair the regeneration potential, which leads to the vulnerability of aging intestinal epithelium to damage factors such as drugs and pathogens. The increased mtROS level resulting from mitochondrial dysfunction was found in Fut2-deficient ISC in this study, which can also be the driver of inflammation in the aging gut by inducing oxidative stress and activation of NLR family pyrin domain containing 3 (NLRP3) inflammasome [51,52]. Furthermore, mitochondrial alterations were demonstrated to correlate to early stage of cancer progression [52,53]. Identifying whether Fut2-deficiency-induced mitochondrial dysfunction in ISC could lead to tumorigenesis of CRC may be interesting in future research.

Finally, we attempted to explore the key protein targets of Fut2 in ISC aging and identify 2 mitochondrial-function-related proteins Asah2 and Npc1. Since protein glycosylation profoundly affects protein traits such as stability and function [54], we found that loss of α1,2-fucosylation modifications on these 2 proteins impairs the respiratory complex and mitophagy in ISC. Asah2 is located on the mitochondrion, Golgi apparatus membrane, and cell membrane, hydrolyzing sphingolipid ceramides into sphingosine and free fatty acids. Asah2 dysfunction results in the accumulation of sphingolipid, which is demonstrated to induce decreased electron transport chain activity and ATP production, fragmentation of mitochondria, ROS production, and chronic oxidative stress in obesity and type 2 diabetes [27,55,56], which is similar to our findings in ISCs. Npc1 is located on the lysosome membrane and functions to mediate cholesterol transport. In addition to the result in proteolysis disorder in the lysosome, Npc1 dysfunction was demonstrated to be associated with the fusion of lysosome and intracellular vesicles, which further affects autophagy and mitophagy [57–59]. In addition, abnormal Npc1 activates downstream mTOR complex, which is an important driver of ISC aging [29,60]. Our findings indicate the critical role of protein fucosylation in regulating mitochondrial function, and more glycomics studies are warranted for our comprehensive understanding.

As the significance of Fut2-mediated α1,2-fucosylation in ISC aging was revealed, therapeutic strategies targeting fucosylation may be applicable regarding some age-related intestinal diseases in future studies. Through the salvage pathway, exogenous fucose supplements could promote the fucosylation process. Fucose was also demonstrated to increase the intestinal epithelial fucosylation level in colitis and ameliorate the inflammation [38]. Moreover, fucose can serve as a prebiotic to regulate the composition of gut microbiota, which is an important driver of fucosylation as mentioned above [38,61]. In addition, targeting the gut bacteria may be beneficial. For example, SFB is a commensal bacterium that could induce α1,2-fucosylation in the intestinal epithelium, and previous studies also reported its immune regulation and anti-inflammatory effects in the small intestine [12,62]. Therefore, SFB may be a potential candidate. Finally, a recent study reported a novel inhibitor of Fut8 that could inhibit the malignance of CRC cells; therefore, we could assume that novel agonists of Fut2 may also be developed for Fut2-deficiency-induced intestinal disorders.

**Fig. 8.**
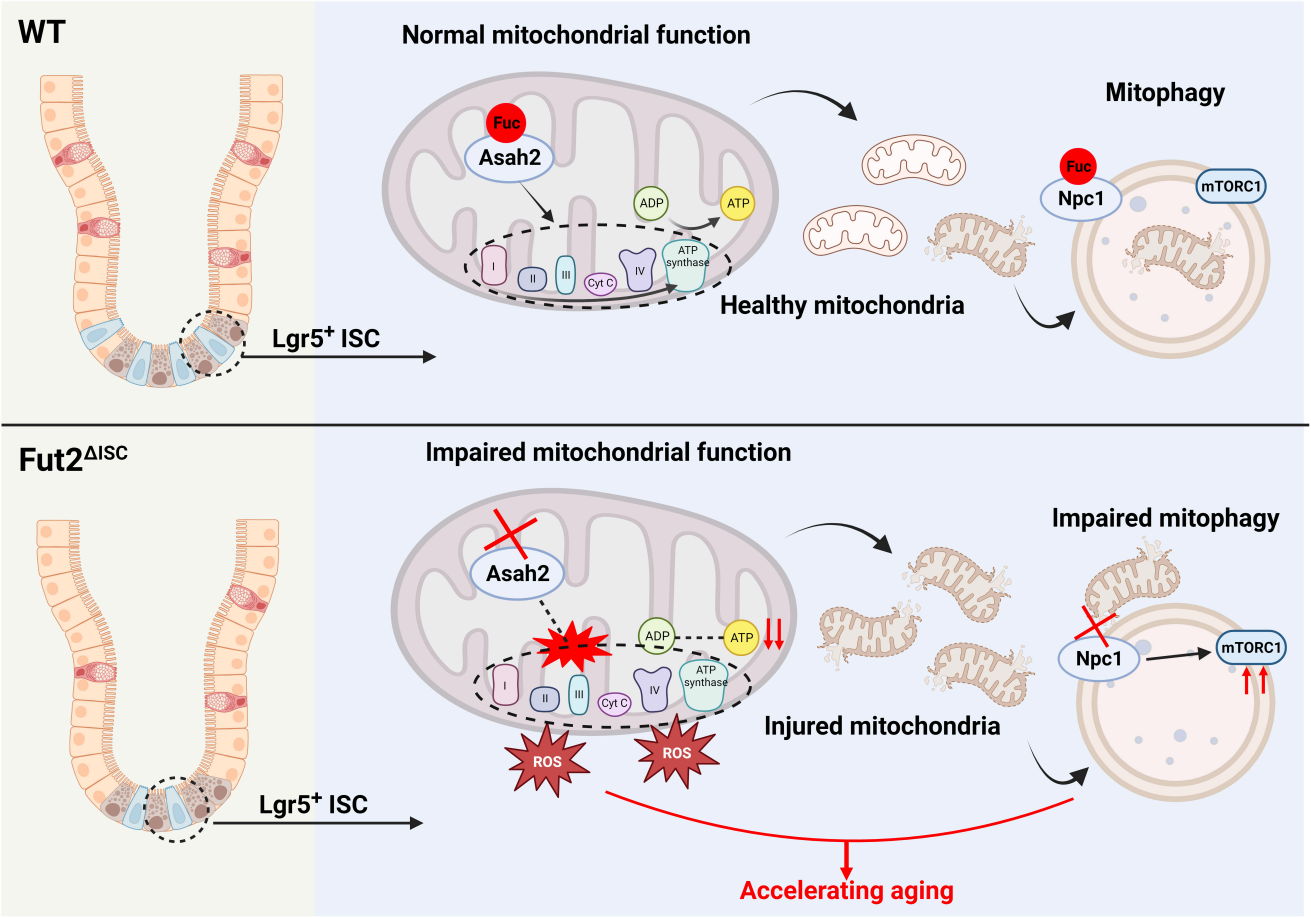
The mechanism of Fut2-deficiency-induced facilitation of ISC aging. Fut2 knockout resulted in loss of α1,2-fucosylation on Asah2 and Npc1, which impaired respiratory complexes and mitophagy, and therefore suppressed ATP production and promoted ROS production. These mitochondrial dysfunctions promoted stemness impairment and ISC aging. This figure is illustrated using BioRender (www.biorender.com).

In conclusion, we demonstrated that deficiency of Fut2-mediated α1,2-fucosylation promotes ISC aging, mainly through Asah2-mediated respiratory complexes and Npc1-mediated mitophagy impairment, which induces mitochondrial dysfunction (Fig. 8). This study provides novel views on the mechanism of intestinal aging and the treatment of aging-related intestinal diseases.

## Materials and Methods

### Clinical specimens

Colon specimens were collected through colonoscopy biopsy with written informed consent from all patients, and their information was anonymized. The approval (ID: 2021-0839) of this study was obtained from the Ethics Committee of Union Hospital, Tongji Medical College, Huazhong University of Science and Technology.

### Animal experiments

Lgr5-EGFP-IRES-Cre^ERT2^ transgenic C57BL/6 mice and Fut2^flox/flox^ C57BL/6 mice were bred and crossed to generate Lgr5-EGFP-IRES-Cre^ERT2^/Fut2^flox/flox^ C57BL/6 mice (WT mice). To induce Fut2-gene-specific knockdown in ISCs (Fut2^ΔISC^ mice), Lgr5-EGFP-IRES-Cre^ERT2^/Fut2^flox/flox^ C57BL/6 mice were daily intraperitoneally injected with 2 mg of tamoxifen (Sigma-Aldrich, T5648) dissolved in 100 μl of corn oil for 5 d. Figure S1 shows the Fut2 expression in ISCs of WT and Fut2^ΔISC^ mice. All mice were bred under the specific-pathogen-free condition in the experimental animal center of Huazhong University of Science and Technology.

To induce aging, WT and Fut2^ΔISC^ mice were fed to 18 months old. In addition, 12-week-old mice were intraperitoneally injected with d-gal (800 mg/kg per day; Sigma-Aldrich, G-0750) for 12 weeks to establish oxidative-stress-induced accelerated aging model referred to previous studies [63,64]. The approval (ID: 2020-2529) of animal experiments in the herein study was obtained from the Animal Experimentation Ethics Committee of Huazhong University of Science and Technology.

### Histological analysis

Tissues were fixed with 4% paraformaldehyde and embedded by paraffin for serial section and H&E staining. The villus height (defined as the length from the villus tip to the crypt–villus junction) and crypt depth (defined as the length from the crypt–villus junction to the bottom of the crypt) of the ileum were measured using ImageJ software.

### Crypts isolation and organoid culture

The ileum was isolated from mice and washed with cold phosphate-buffered saline (PBS). Then, we cut the tissues into sections and incubated them with 2.5 mM EDTA on ice for 40 min. We removed the supernatant and suspended the tissues with ice-cold PBS containing 0.1% bovine serum albumin (BSA). After shaking, we used a 70-μm cell strainer to filter the samples and centrifuged at 290*g* for 5 min. Next, we removed the supernatant, washed the samples with ice-cold PBS containing 0.1% BSA, and centrifuged at 200*g* for 5 min to remove single cells and get crypts. Crypts were suspended with IntestiCult Organoid Growth Medium (STEMCELL Technologies, 06000) and Matrigel Matrix (Corning, 354277). Fifty microliters of cell suspension was plated into the center of 24-well plates to form a dome. Organoids were cultured at 37 °C under 5% CO_2_. ImageJ software was used to measure the surface areas and budding number of organoids in several random fields. Images were obtained by an inverted microscope (Carl Zeiss).

To establish an in vitro model of oxidative-stress-induced aging, organoids derived from 12-week-old mice were treated with 500 μM hydrogen peroxide (H_2_O_2_; Sigma-Aldrich, 88597) for 12 h. CCCP (10 μM; Selleck, S6494) treatment for 4 h was used to activate mitophagy. Ten micrograms of Asah2 recombinant protein (MedChemExpress, HY-P76735) and 250 nM mTOR inhibitor Torin1 (MedChemExpress, HY-13003) were used to treat organoids to confirm the role of Asah2 and Npc1 fucosylation.

### Immunostaining

For tissue IF staining, ileal sections were dewaxed and rehydrated before antigen heat retrieval. Then the sections were permeabilized and blocked with 0.3% Triton X-100 and donkey serum, respectively, for primary antibodies incubated overnight at 4 °C. Next, incubating the samples with rhodamine-conjuncted UEA-I for detection of α1,2-fucosylation (UEA-I; Vector Laboratories, RL-1062) or secondary antibodies. Cell nuclei were stained by 4′,6-diamidino-2-phenylindole (DAPI). Images were obtained by a fluorescence microscope (Carl Zeiss). The images were analyzed with ImageJ software. An streptavidin-biotin complex-alkaline phosphatase (SABC-AP) kit for rabbit immunoglobulin G (Boster Biological Technology, SA1052) was used for IHC analysis according to the manufacturer’s instructions. Briefly, paraffin sections were dewaxed and rehydrated before antigen heat retrieval. The sections were blocked with 0.5% BSA, incubated with primary antibodies, corresponding biotin-conjunct secondary antibodies and SABC-AP reagent, and colored with 5-bromo-4-chloro-3-indolyl phosphate/nitroblue tetrazolium chloride (BCIP/NBT) reagent. The images were analyzed with the IHC Profiler plugin of ImageJ software.

IF quantification analysis of mitochondrial respiratory complex proteins was performed as previously described [24,25]. Marker proteins of respiratory complexes were costained with mitochondrial marker Tomm20. Mean fluorescence intensity values of respiratory complex proteins in crypts were obtained by ImageJ software and normalized to mean fluorescence intensity values of Tomm20. *z* score of respiratory complex proteins was calculated, and crypts with *z* scores of <−2 were defined as respiratory complex deficient.

For organoid IF staining, organoids were collected and resuspended in 4% paraformaldehyde for fixing, and the following steps are similar to tissue section staining. Organoids were also embedded in agarose for serial section in a part of IF experiments for mitophagy analysis. Colocalization analysis was performed using Image-Pro Plus software.

The antibodies used for immunostaining are shown in Table S1.

### SA-β-gal analysis

Senescence cells were detected by an SA-β-gal staining kit (Beyotime Biotechnology, C0602) following the manufacturer’s instructions. Briefly, organoids or frozen sections of tissues were fixed and incubated with a staining solution overnight at 37 °C without CO_2_. Then samples were washed with PBS and observed by a light microscope (Olympus).

### EdU assay

EdU assay was performed to analyze cell proliferation in organoids using an EdU cell proliferation kit (Beyotime Biotechnology, C0078) following the manufacturer’s instruction. Briefly, 10 μM EdU reagent was added to organoids and incubated for 2 h. Then, cells were fixed, permeabilized, and incubated with the reaction solution. Cell nuclei were stained by DAPI.

### RNA extraction and real-time PCR

Total RNA of tissues and organoids was extracted using an RNeasy RNA Isolation Kit (Beyotime Biotechnology, R0024) following the manufacturer’s instructions and applied for reverse transcription using a HiScript II 1st Strand cDNA Synthesis Kit (Vazyme, R211-01). The program of real-time PCR was run on a Roche LightCycler R480 system (Roche) with a Power SYBR Green Master Mix (Thermo Fisher Scientific, 4367659). The 2^−ΔCT^ method was used to calculate relative gene expression normalized to glyceraldehyde-3-phosphate dehydrogenase (GAPDH). Table S2 shows the primers that were used in this study.

### Oxidative stress analysis

Ileum crypts were isolated as described above and examined CAT, GSH-PX, MDA, and SOD activities using a corresponding assay kit (Nanjing Jiancheng, A007-1, A005-1, A003-1, and A001-3) to analyze the oxidative stress level following the manufacturer’s instructions.

### ISC sorting

For ISC isolation, crypts derived from WT and Fut2^ΔISC^ mice were dissociated with TrypLE Express (Gibco, 12604) at room temperature for 10 min to get single cells. Cells were then incubated with GFP antibody for 30 min at 37 °C and suspended in Dulbecco’s modified Eagle’s medium/F-12 medium (Gibco, 11330) for fluorescence-activated cell sorting to isolate GFP^+^ cells. A MoFlo XDP cell sorter (Beckman Coulter) was used for fluorescence-activated cell sorting.

### Tandem mass tag-labeled quantitative proteomics and N-linked glycosylation modification quantitative proteomics

For TMT-labeled quantitative proteomics, ISC samples were sonicated and centrifuged at 12,000*g* at 4 °C for 10 min. After digested by trypsin and labeled by tandem mass tags (TMTs), the samples were fractionated by high pH reverse-phase high-performance liquid chromatography and analyzed by liquid chromatography-tandem mass spectrometry. The result data were processed using the MaxQuant search engine (v.1.6.15.0).

For N-linked glycosylation modification quantitative proteomics, samples were fractionated by high-performance liquid chromatography. Tryptic peptide solution was loaded onto the column. Glycopeptides were eluted with 0.1% trifluoroacetic acid, 50 mM ammonium bicarbonate, and 50% acetonitrile (ACN) 2 times, and the samples were dried. Then, the samples were redissolved in 50 mM ammonium bicarbonate solution and digested at 37 °C overnight with 2 μl of peptide *N*-glycosidase F glycosidase. Finally, deglycopeptides were desalted by C18 Zip Tips following the manufacturer’s instructions, and the samples were dried for liquid chromatography-tandem mass spectrometry analysis.

UniProt-GOA database was used to derive GO annotation, and the KEGG database was used to derive pathway annotation.

### TEM analysis

The ultrastructure of ISCs was observed by TEM. Ileum tissues were fixed with 2.5% glutaraldehyde, postfixed with 1% osmium tetroxide, rinsed with PBS, dehydrated in increasingly graded alcohols, and embedded in Epon. Samples were cut into ultrathin sections using an ultramicrotome and examined by a Hitachi TEM system (Hitachi).

### ROS detection

DHE (Beyotime Biotechnology, S0063) was used to detect total ROS in cells. Crypts, organoids, or frozen sections of tissues were incubated with 5 μM DHE for 30 min at 37 °C, washed with PBS, and analyzed using a flow cytometer or fluorescence microscope.

MitoSOX Red mitochondrial superoxide indicator (Invitrogen, M36009) was used to analyze mtROS. Crypts or organoids were incubated with 500 nM MitoSOX for 30 min at 37 °C, washed with PBS, and analyzed using a flow cytometer or fluorescence microscope.

### MMP analysis

JC-1 MMP assay kit (Beyotime Biotechnology, C2003) was used to analyze MMP following the manufacturer’s instructions. Organoids were incubated with JC-1 working solution at 37 °C for 20 min, then washed with washing buffer, and observed using a fluorescence microscope. The green JC-1 monomers indicate low MMP, while the red JC-1 aggregates indicate high MMP.

### Protein extraction, lysosome fractions enrichment, and Western blot analysis

Radioimmunoprecipitation assay lysis buffer containing 1% phenylmethyl sulfonyl fluoride protease inhibitor and phosphatase inhibitor was used to harvest the total protein of organoids. A bicinchoninic acid (BCA) kit (Servicebio, G2026) was used to detect the protein concentration. To investigate the status of mitophagy, organoids were treated with 10 μM chloroquine (Selleck, S6999) for 1 h after CCCP treatment, and lysosome fractions were enriched from organoids using the Lysosome Enrichment Kit for Tissue and Cultured Cells (Thermo Fisher Scientific, 89839) following the manufacturer’s instructions. Samples were separated by SDS-polyacrylamide gel electrophoresis, transferred to polyvinylidene fluoride membranes, blocked by 5% skimmed milk, and then incubated with primary antibodies and corresponding horseradish-peroxidase-conjunct secondary antibodies. The images of blots were obtained by a chemiluminescence imaging system (UVP) using an ECL chemiluminescence detection kit (Vazyme, E411). Table S1 shows the antibodies that were used in this study.

### Activities of respiratory chain complexes

The activities of mitochondrial respiratory chain complexes I to V of crypts derived from mice were analyzed using a CheKine micro mitochondrial complex activity assay kit (Abbkine, KTB1850, KTB1860, KTB1870, KTB1880, and KTB1890) following the manufacturer’s instructions.

### ATP assay

An ATP assay kit (Beyotime Biotechnology, S0026) was used to detect the ATP level in organoids following the manufacturer’s instructions. Briefly, the cell lysate was centrifuged at 4 °C 12,000*g* for 10 min, and the supernatant was added into the working solution in a light-tight 96-well plate. The luminescence was analyzed with a luminometer, and the ATP concentration was calculated according to the standard curve.

### UEA-I chromatography

UEA-I chromatography was performed as described before [65]. UEA-I chromatography was used to examine the alteration of protein α1,2-fucosylation modification. The proteins with α1,2-fucosylation modification were enriched by agarose-conjunct UEA-I and then detected by Western blot. Briefly, cells were lysed with ice-cold cell lysis buffer for Western blot and immunoprecipitation (Beyotime Biotechnology, P0013) to harvest the total protein. The protein concentration was confirmed through the BCA method (Servicebio, G2026). Cell lysate (500 μg) was mixed with 50 μl of agarose-bound UEA-I (Vector Laboratories, AL-1063) and incubated overnight at 4°C with rotation for α1,2-fucosylated proteins enrichment. Then, the samples were washed 3 times with lysis buffer and centrifuged at 1,000 rpm for 2 min at 4 °C. UEA-I enriched proteins were extracted by 100 °C heating for 10 min with 5× SDS-polyacrylamide gel electrophoresis loading buffer and centrifuging at 1,000 rpm for 2 min.

### Organoid transfection

To verify the effects of α1,2-fucosylation modification on functions of Asah2, Npc1, and Bsg that were screened through N-linked glycosylation modification quantitative proteomics, SDM plasmids were constructed on cloning vector pcDNA3.1 (Tsingke Biotechnology) and were transfected to organoids to replace the asparagine modification site of selective proteins with glutamine to inhibit α1,2-fucosylation on the site. Organoid transfection was performed as described before with little modifications [66]. Briefly, organoids were trypsinized with TrypLE Express (Gibco, 12604) for 10 min at room temperature, centrifuged at 300*g* for 5 min at 4 °C, resuspended with culture medium, and plated in 48-well plates. Then, the DNA-Lipofectamine 3000 reagent (Invitrogen, L3000001) complexes were prepared with Opti-MEM medium (Gibco, 31985070) and added to the cells following the manufacturer’s instructions. The plates were centrifuged and incubated at 37 °C for 4 h. Then, the cells were collected and replated in Matrigel.

### Statistical analysis

Statistical analysis was performed using SPSS 25 and visualized by GraphPad Prism software (version 7.0). All experiments were performed at least in triplicate, and data were presented as the means ± SEM. Two-tailed *t* tests or one-way analysis of variance (ANOVA) followed by Tukey’s post hoc test were used to analyze the differences between groups. Statistically significant was considered as *P < 0.05, **P < 0.01, and ***P < 0.001.

## Data Availability

Data will be made available on request.
