# Supplementary material for: Fut2 Deficiency Promotes Intestinal Stem Cell Aging by Damaging Mitochondrial Functions via Down-Regulating α1,2-Fucosylation of Asah2 and Npc1
Source: Research (Wash D C). 2024 Mar 27;7:0343. doi: 10.34133/research.0343 (PMC10976588; doi:10.34133/research.0343)
Supplement: Supplementary 1 — Tables S1 and S2 Figs. S1 to S7 [file research.0343.f1.docx]

**Supplementary Materials**

**Supplementary Table 1. Antibodies used for immunostaining and western blot.**

| Antibody | Brand | Catalog number |
| --- | --- | --- |
| Fut2 | Immunoway | YT1801 |
| Fut2 | Abclonal | A5721 |
| Olfm4 | Cell Signaling Technology | 39141 |
| β-catenin | Abclonal | A19657 |
| Wnt3 | Abcam | ab219412 |
| GFP | Abcam | ab290 |
| Ki67 | Servicebio | GB111141 |
| ZO1 | Genetex | GTX636491 |
| Claundin1 | Genetex | GTX54539 |
| Tomm20 | Abcam | 56783 |
| PGC1α | Abclonal | A12348 |
| Drp1 | Abclonal | A2586 |
| Vdac1 | Abclonal | A19707 |
| Timm23 | Abclonal | A8688 |
| Mfn1 | Abclonal | A9880 |
| Mfn2 | Abclonal | A19678 |
| Tfam | Abclonal | A3173 |
| Cycs | Abclonal | A4912 |
| ACTB | Abclonal | AC026 |
| Atpb | Abcam | ab14730 |
| Mt-co1 | Abcam | ab203912 |
| Ndufb8 | Abcam | ab192878 |
| Uqcrfs1 | Abcam | ab191078 |
| Lamp1 | Abcam | ab208943 |
| LC3B | Cell Signaling Technology | 83506 |
| Phospho-Ubiquitin-Ser65 | Cell Signaling Technology | 62802 |
| Pink1 | Genetex | GTX107851 |
| GAPDH | Abclonal | AC001 |
| Npc1 | Cell Signaling Technology | 5058 |
| Asah2 | Genetex | GTX55519 |
| Bsg | Abclonal | A4310 |
| Phospho-mTOR-Ser2448 | Cell Signaling Technology | 5536 |
| mTOR | Abclonal | A2445 |
| Alexa Fluor 488-donkey anti goat IgG | Antgene | ANT025 |
| Alexa Fluor 488-donkey anti rabbit IgG | Antgene | ANT024 |
| Alexa Fluor 594-donkey anti rabbit IgG | Antgene | ANT030 |
| Alexa Fluor 647-donkey anti rabbit IgG | Antgene | ANT032 |
| Alexa Fluor 488-donkey anti mouse IgG | Antgene | ANT023 |
| Alexa Fluor 594-donkey anti mouse IgG | Antgene | ANT029 |
| HRP-goat anti rabbit IgG | Antgene | ANT020 |
| HRP-goat anti mouse IgG | Antgene | ANT019 |

**Supplementary Table 2. Sequences of primers used for qPCR.**

| Gene | Forward primer (5’-3’) | Reverse primer (5’-3’) |
| --- | --- | --- |
| Fut2 | GGGCACGCTATTCATCTCCA | AGCCCTCAATACCATTGCCC |
| p15^INK4b^ | CAATCCAGGTCATGATGATGGG | TGCACAGGTCTGGTAAGGGT |
| p21^CIP1^ | ACGGTGGAACTTTGACTTCGT | GAGTGCAAGACAGCGACAAG |
| IL-1β | CTCAACTGTGAAATGCCACCT | AAGGTCCACGGGAAAGACAC |
| IL-6 | ACAAAGCCAGAGTCCTTCAGAG | CCACTCCTTCTGTGACTCCA |
| Lgr5 | CCTACTCGAAGACTTACCCAGT | GCATTGGGGTGAATGATAGCA |
| Olfm4 | CAGCCACTTTCCAATTTCACTG | GCTGGACATACTCCTTCACCTTA |
| Ascl2 | AAGCACACCTTGACTGGTACG | AAGTGGACGTTTGCACCTTCA |
| Wnt3 | CTCGCTGGCTACCCAATTTG | CTTCACACCTTCTGCTACGCT |
| Axin2 | TGACTCTCCTTCCAGATCCCA | TGCCCACACTAGGCTGACA |
| Ctnnb1 | ATGGAGCCGGACAGAAAAGC | CTTGCCACTCAGGGAAGGA |
| GAPDH | CCCAGCTTAGGTTCATCAGG | CAAATCCGTTCACACCGACC |


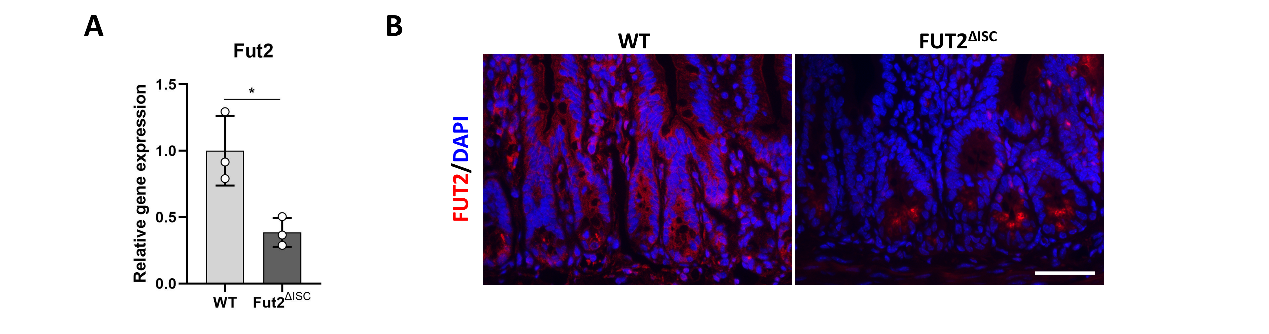
 **Supplementary Fig. 1. Validation of Fut2 knockdown.** (A) Gene expression of Fut2 in ISCs of WT and Fut2^ΔISC^ mice. (B) Fluorescence in Situ Hybridization of Fut2 in ileal sections of WT and Fut2^ΔISC^ mice (Scale bar, 100 μm).


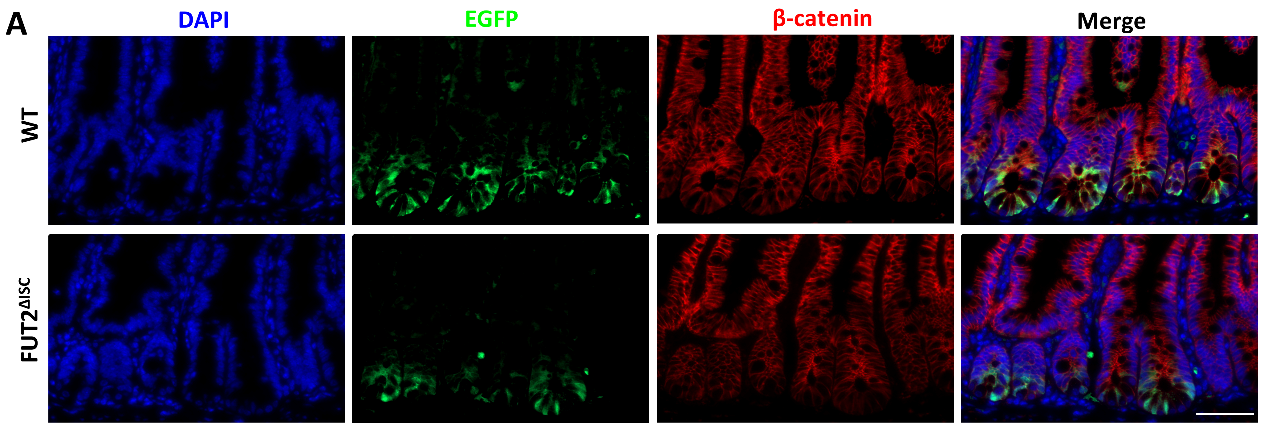


**Supplementary Fig. 2. β-catenin expression decreased ISCs of Fut2^ΔISC^ mice.** (A) EGFP and β-catenin staining in ileal sections of WT and Fut2^ΔISC^ mice (Scale bar, 100 μm).


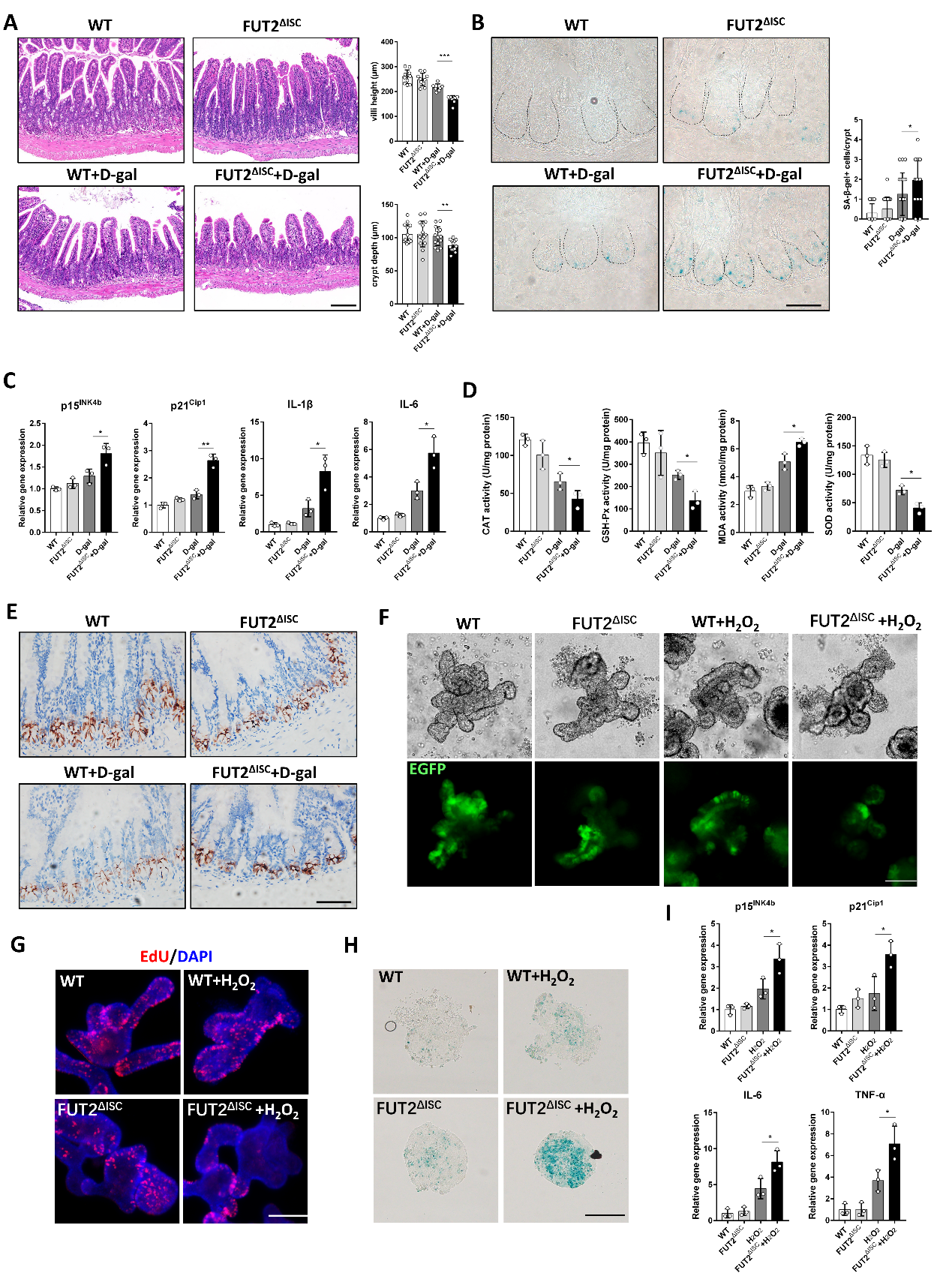


**Supplementary Fig. 3.** **Fut2 deficiency of ISCs promotes the D-gal induced senescence in mice ileum.** (A) HE analysis of ileal sections from WT, FUT2^ΔISC^, WT+D-gal, and FUT2^ΔISC^+D-gal mice and statistic of villi height and crypt depth (Scale bar, 100 μm). (B) SA-β-gal detection in ileal tissues of WT, FUT2^ΔISC^, WT+D-gal, and FUT2^ΔISC^+D-gal mice. Blue staining indicates senescence cells and the dotted line indicates crypt (Scale bar, 100 μm). (C) Expression of senescence marker and senescence-associated secretory phenotypes genes in crypts of WT, FUT2^ΔISC^, WT+D-gal, and FUT2^ΔISC^+D-gal mice. (D) The activity of CAT, GSH-Px, MDA, and SOD in crypts. (E) Olfm4 expression in ileal sections detected by IHC assays (Scale bar, 100 μm). (F) Images of H_2_O_2_-treated organoids derived from WT and FUT2^ΔISC^ mice and IF staining of EGFP (Scale bar, 100 μm). (G) EdU analysis of H_2_O_2_-treated WT and FUT2^ΔISC^ organoids (Scale bar, 100 μm). (H) SA-β-gal analysis in H_2_O_2_ treated WT and FUT2^ΔISC^ organoids (Scale bar, 100 μm). (I) Gene expression of senescence marker and senescence-associated secretory phenotypes in H_2_O_2_ treated WT and FUT2^ΔISC^ organoids.


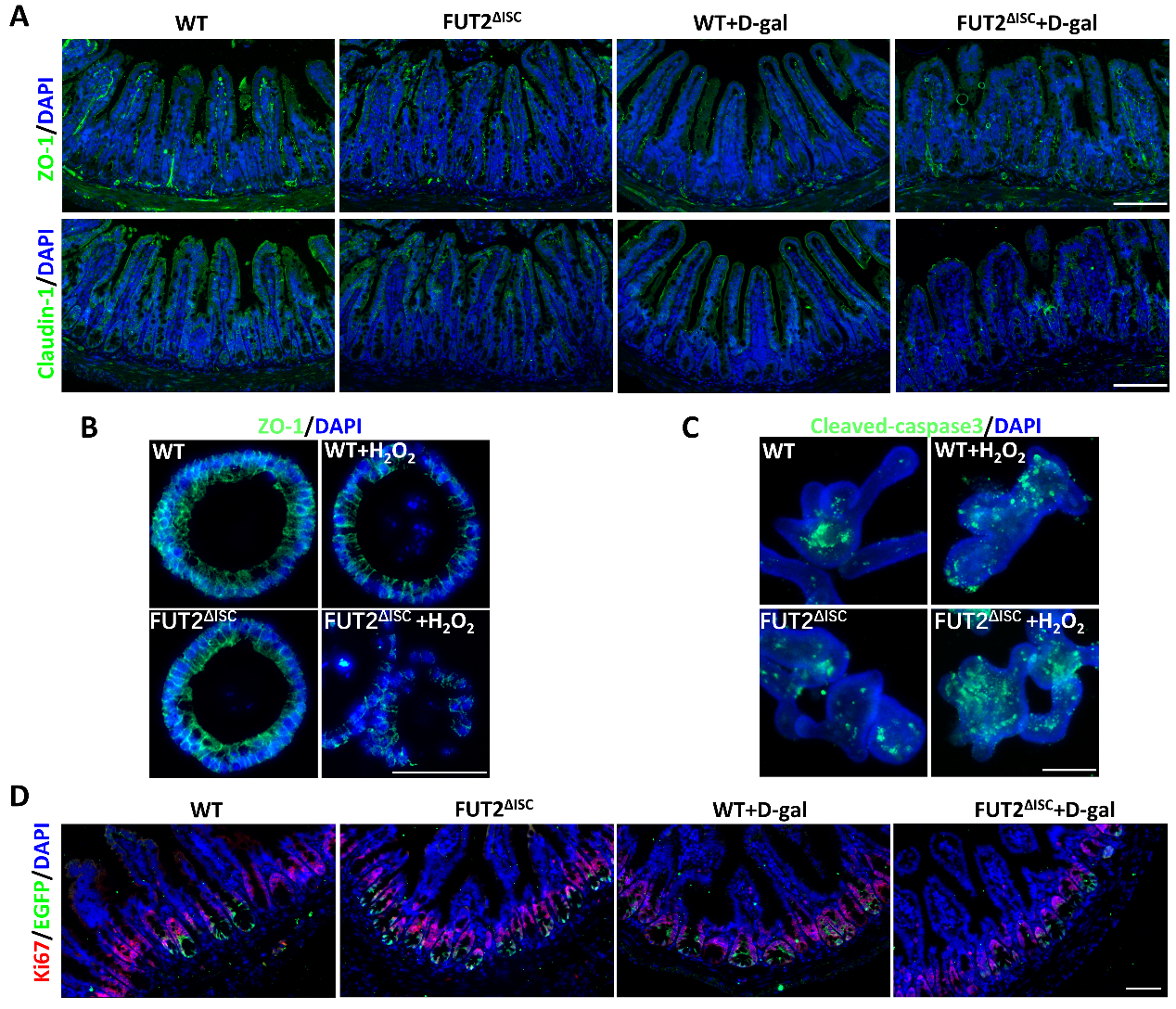


**Supplementary Fig. 4.** **Fut2 deficiency ISCs were more vulnerable to oxidative stress damage.** (A) IF analysis of ZO-1 and claudin-1 in ileal sections from WT, FUT2^ΔISC^, WT+D-gal, and FUT2^ΔISC^+D-gal mice. (B-C) ZO-1 and Cleaved-caspase 3 staining in WT, FUT2^ΔISC^, WT+H_2_O_2_, and FUT2^ΔISC^+H_2_O_2_ organoids. (D) EGFP and Ki67 staining in ileal sections from WT, FUT2^ΔISC^, WT+D-gal, and FUT2^ΔISC^+D-gal mice (Scale bar, 100 μm).


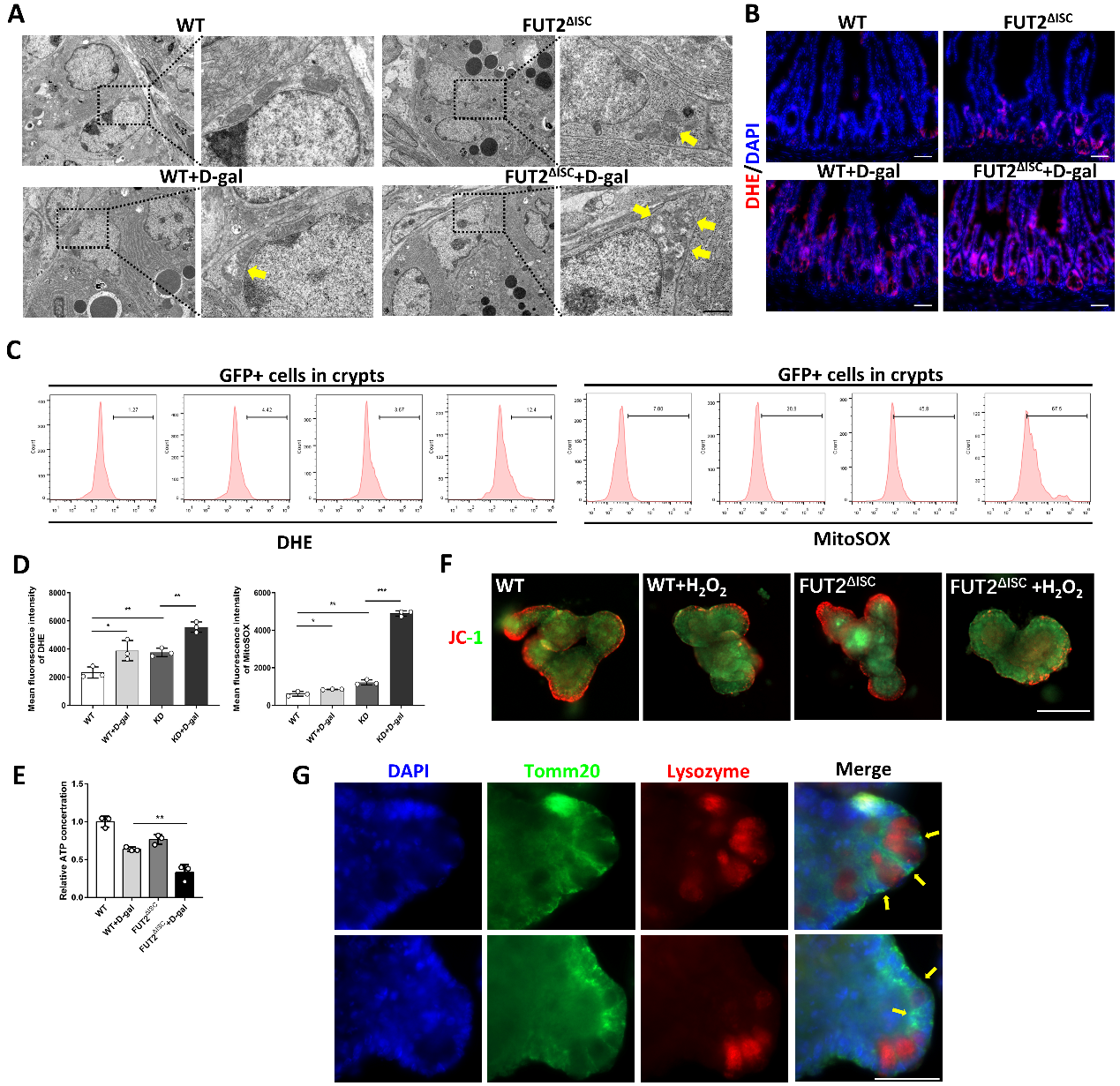


**Supplementary Fig. 5. Mitochondria were more damaged in ISC of FUT2^ΔISC^+D-gal mice.** (A) TEM analysis of mitochondria in ISCs of WT, FUT2^ΔISC^, WT+D-gal, and FUT2^ΔISC^+D-gal mice. Yellow arrows indicate damaged mitochondria (Scale bar, 5 μm and 1μm). (B) ROS level in ileal sections indicated by DHE staining. (C-D) Flow cytometry detection of DHE-indicated ROS and MitoSOX-indicated mtROS in ISCs of WT, FUT2^ΔISC^, WT+D-gal, and FUT2^ΔISC^+D-gal mice. (E) ATP concentration in ISCs of aged WT and FUT2^ΔISC^ mice, and ISCs of WT, FUT2^ΔISC^, WT+D-gal, and FUT2^ΔISC^+D-gal mice. (F) JC-1-indicated MMP in WT and FUT2^ΔISC^ organoids treated by H_2_O_2_ (Scale bar, 100 μm)**_._** (G) Lysozyme and Tomm20 staining in organoids derived from aged WT and FUT2^ΔISC^ mice. Yellow arrows indicate ISCs (Scale bar, 30 μm).


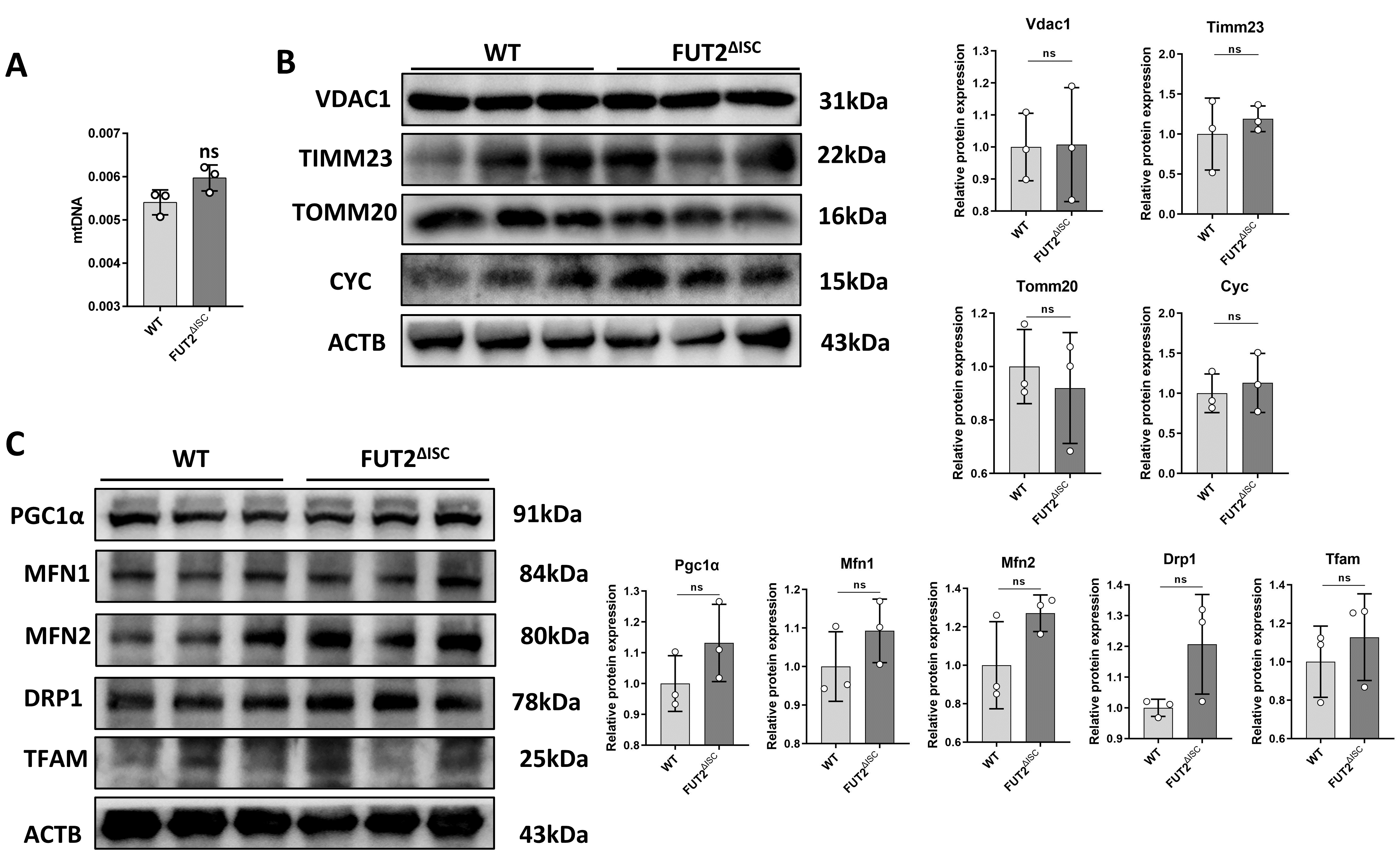


**Supplementary Fig. 6.** **Mitochondrial content and dynamics protein expression were not altered in the ISC of FUT2^ΔISC^ mice.** (A) mtDNA level in aged WT and FUT2^ΔISC^ mice. (B and C) Western blot analysis of mitochondria proteins.


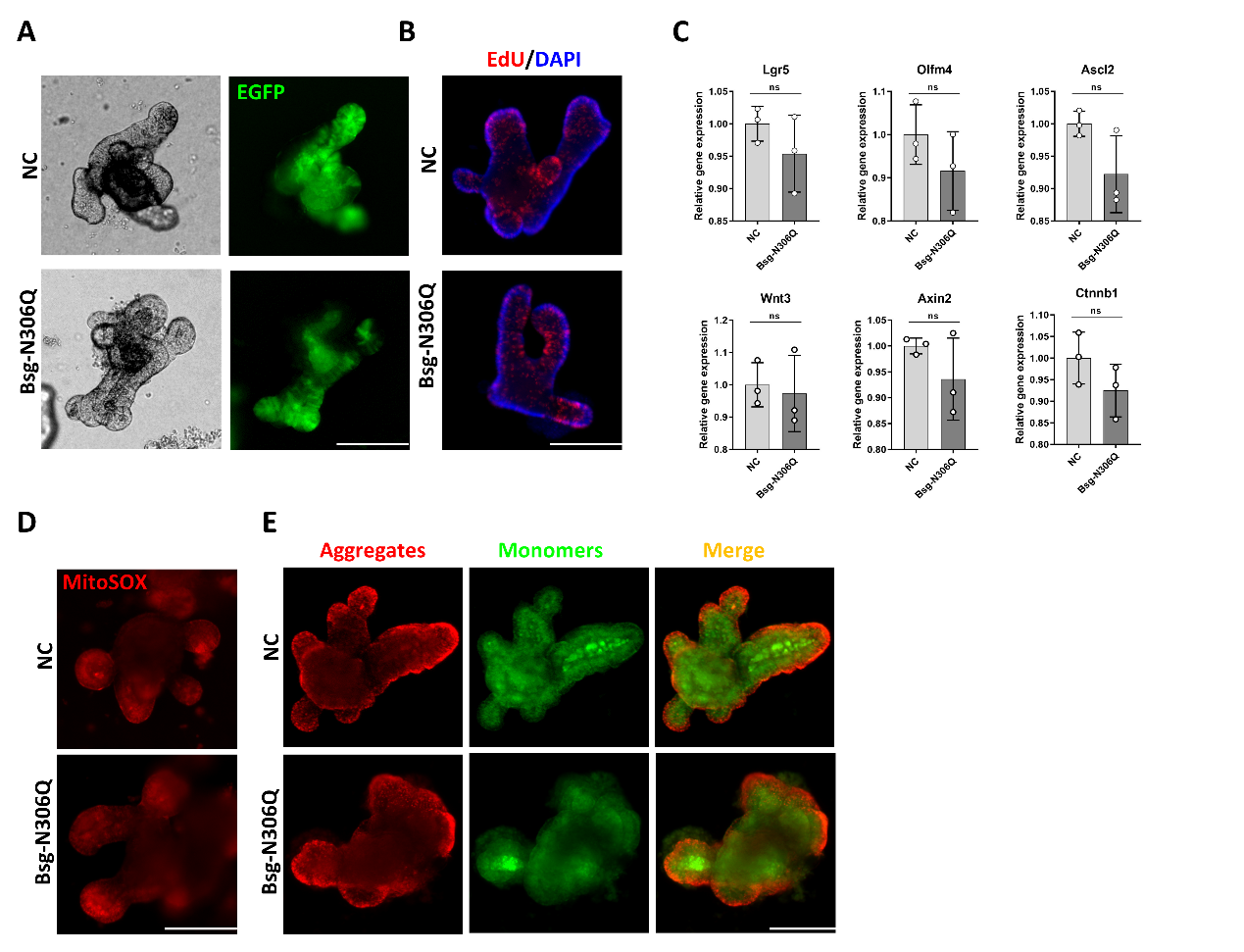


**Supplementary Fig. 7.** **Mutant of Bsg did not significantly affect stemness and mitochondrial functions of organoids.** (A) Images of WT, Bsg-N3306Q organoids, and IF analysis of EGFP (Scale bar, 100 μm). (B) EdU assays of WT and Bsg-N3306Q organoids. (C) PCR analysis of stemness markers in WT and Bsg-N3306Q organoids. (D) mtROS in WT and Bsg-N3306Q organoids. (E) MMP in WT and Bsg-N3306Q organoids (Scale bar, 100 μm).
